# Novel *N*-4-(5-Amino-7-substituted-triazolotriazinepiperazin-1-yl) Norfloxacin Analogues Exhibit Potent and Selective Anticancer Activity via Topoisomerase Inhibition, Cell-Cycle Arrest, and Apoptosis in A 431 Skin Carcinoma Cells

**DOI:** 10.3390/ph19050657

**Published:** 2026-04-22

**Authors:** Ahmed M. El-Saghier, Amany M. Hamed, Laila Abosella, Stefan Bräse, Hossameldin A. Aziz

**Affiliations:** 1 Chemistry Department, Faculty of Science, Sohag University, Sohag 282524, Egypt; amanymohamed@science.sohag.edu.eg; 2 Pharmaceutical Chemistry Department, Faculty of Pharmacy-Aljmail, Sabratha University, Sabratha 250, Libya; 3 Institute for Biological and Chemical Systems, Karlsruhe Institute of Technology, 76131 Karlsruhe, Germany; 4 Pharmaceutical Chemistry Department, Faculty of Pharmacy, New Valley University, New Valley 72511, Egypt; hossamaziz85@pha.nvu.edu.eg; 5 Pharmaceutical Chemistry Department, Faculty of Pharmacy, New Valley National University, New Valley 72511, Egypt

**Keywords:** skin carcinoma, topoisomerase I/II, cell-cycle arrest, apoptosis, norfloxacin

## Abstract

**Background**: Skin carcinoma is among the most common cancers globally, necessitating the urgent development of innovative chemotherapeutic drugs that exhibit great selectivity and diminished toxicity towards normal cells. This study assessed a series of recently synthesized compounds (**4–15**) for screening their anticancer efficacy against A 431 human skin carcinoma cells to find effective and selective treatment candidates. **Methods**: Compounds were synthesized via a one-pot, three-component reaction. Cytotoxicity was evaluated using the MTT assay, with 5-fluorouracil (5-FU) as the reference standard, which exhibited potent activity against A 431 skin cancer cells. The activity of these drugs against normal BJ cells was evaluated, with mechanistic investigations encompassing topoisomerase I/II enzyme inhibition, cell-cycle analysis, and Annexin V–FITC/PI apoptosis assays. **Results**: Most compounds exhibited dose-dependent cytotoxicity, with compound **14** demonstrating the highest potency (IC_50_ = 76.7 µg/mL), exceeding that of 5-FU (IC_50_ = 83.7 µg/mL) while preserving selectivity for BJ cells. Compound **14** exhibited moderate inhibition of topoisomerases I and II (IC_50_ values of 17.5 and 17.3 µM), as confirmed by docking studies. Flow cytometry indicated G0/G1 phase arrest (64.09% vs. 58.18% in control), while apoptosis assays confirmed induction of both early and late apoptosis, accompanied by significant necrosis. **Conclusions**: Compound **14** is the most efficacious and selective drug in this series, functioning through topoisomerase inhibition, G0/G1 cell cycle arrest, and apoptosis, thereby representing a strong candidate for subsequent preclinical research.

## 1. Introduction

The widespread toxicity of conventional cancer chemotherapeutic medications continues to make it extremely challenging to provide effective cancer treatment, even though our understanding of the molecular pathways that underlie carcinogenesis has been enhanced. Thus, medicinal chemists worldwide must find and create new cancer treatments. Cancer ranks second in global mortality behind cardiovascular disease [1,2,3,4].

Although therapy options have improved, there is still a need for new, selective anticancer drugs that target skin cancer while avoiding or significantly reducing harm to healthy tissues. Low selectivity and dose-related adverse effects are common problems with traditional chemotherapeutics such as antimetabolites [5,6,7].

Pharmacological scaffolds with high potency and safety have gained popularity in recent decades. Fluoroquinolone derivatives are promising. Fluoroquinolone-based medicines inhibit topoisomerase I/II, induce cell cycle arrest, and induce apoptosis, all while being less hazardous than standard chemotherapy medications [6,8]. In parallel, medicinal chemistry research focuses on designing novel compounds that target cancer-specific pathways. Recently discovered thiazolo[3,2-a]pyrimidine compounds inhibit topoisomerase II, killing cancer cells and interrupting the cell cycle [9]. Hybrids of chemotherapeutics used with natural or synthetic topoisomerase II inhibitors may improve efficacy and reduce toxicity [10]. Fluoroquinolone derivatives have been demonstrated to inhibit topoisomerase II in cancer cells mechanistically [4,11]. Recent studies of cancer-active fluoroquinolone analogues have confirmed their ability to interfere with DNA through topoisomerase inhibition, to cause G2/M or G1 cell-cycle arrest, and to initiate apoptotic or necrotic cell death [11]. Moreover, the structure–activity correlations of these derivatives indicate that alterations at the piperazine moiety or other sites can significantly augment their anticancer efficacy [12]. Concurrently, innovative levofloxacin-derived compounds were developed to selectively inhibit human topoisomerase II beta, exhibiting significant cytotoxicity against cancer cells while minimizing effects on normal cells [13]. New norfloxacin analogues demonstrated significant cytotoxicity against HeLa cells, with IC_50_ values in the low-micromolar range, and exhibited favourable ADME (absorption, distribution, metabolism, excretion) characteristics. Furthermore, further norfloxacin derivatives have demonstrated in silico suppression of BCL-2, indicating their potential to induce apoptosis in cancer cells [14].

Norfloxacin, along with other fluoroquinolones, possesses a carboxyl group and a fundamental piperazinyl moiety. The piperazinyl group enhances the molecule’s suitability as a core for various structural modifications, generating new derivatives that can address emerging resistance while augmenting the potency and efficacy of the parent molecule, without compromising its safety profile [15,16].

N-4-piperazinyl substitution of fluoroquinolones enhances their physicochemical properties, augments lipophilicity, elevates efficacy against Gram-positive bacteria, and predominantly preserves activity against Gram-negative pathogens [15]. A variety of N-piperazinyl substituents was synthesized and evaluated for their antibacterial effectiveness in this context [16,17]. Purine analogues possess a significant historical background in anticancer treatment. In depth, a purine nucleoside phosphorylase (PNP) inhibitor has undergone clinical evaluation for T-cell malignancies, such as cutaneous T-cell lymphoma, exhibiting substantial antitumor efficacy by promoting intracellular accumulation of deoxyguanosine triphosphate (dGTP) and inducing apoptosis in lymphoid cells [18,19,20].

Initially developed for leukemia, purine-based PNP inhibitors have motivated medicinal chemists to develop new purine bioisosteres that maintain or enhance biological activity and selectivity. A highly promising strategy is the use of 1,3,5-triazine-based scaffolds, which emulate the purine core and have been thoroughly investigated as preferred structures for anticancer and other therapeutic applications [21,22].

In this setting, triazine-fused heterocycles, such as triazolo triazines, have emerged as adaptable purine isosteres. These frameworks integrate the structural characteristics of purine with enhanced chemical diversity, facilitating the creation of innovative molecules with superior pharmacological properties and less toxicity [23]. Therefore, the present study was designed to synthesize and evaluate a new series of N-4-(5-amino-7-substituted triazolotriazine-piperazinyl) norfloxacin analogues as potential anticancer agents. By integrating the pharmacological advantages of the fluoroquinolone backbone with the purine-mimic triazolotriazine scaffold, we aimed to generate hybrid molecules with improved anticancer activity, selectivity, and mechanistic diversity. To this end, we systematically assessed their cytotoxic effects against A 431 human skin carcinoma cells, evaluated their safety profile in normal BJ fibroblasts, and explored their molecular mechanisms through topoisomerase I/II inhibition assays, cell-cycle analysis, and apoptosis profiling. Ultimately, the goal of this work is to identify potential lead candidates within this new structural class that could serve as the basis for developing safer and more effective treatments for skin cancer.

## 2. Results

### 2.1. Chemistry

**Scheme 1:**
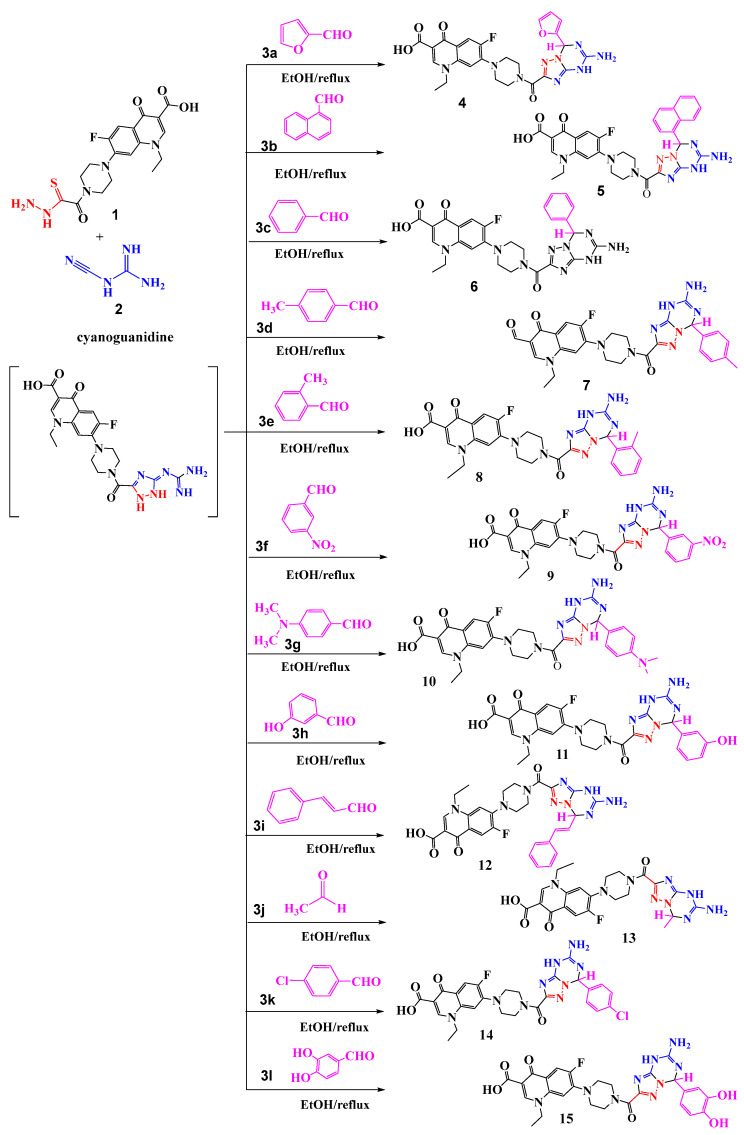
Synthesis of 7-(4-(5-amino-7-substituted-4,7-dihydro-[1,2,4]triazolo[1,5-a][1,3,5]triazine-2-carbonyl)piperazin-1-yl)-1-ethyl-6-fluoro-4-oxo-1,4-dihydroquinoline-3-carboxylic acid derivatives (**4**–**15**).

As part of our ongoing research into the synthesis of novel heterocycles/fluoroquinolone hybrids [24,25,26,27,28,29,30,31,32,33,34,35], herein, a new series of 7-(4-(5-amino-7-substituted-4,7-dihydro-[1,2,4]triazolo[1,5-a][1,3,5]triazine-2-carbonyl)piperazin-1-yl)-1-ethyl-6-fluoro-4-oxo-1,4-dihydroquinoline-3-carboxylic acid compounds were synthesised using a novel, easy method with a one-pot reaction and green solvent (EtOH), saving energy (at room temperature), cost (without catalyst) and time (about 3 h), with no requirements for toxic chemicals and achieving a high yield (79–93%) and meeting the green synthesis requirements. Thiocarbohydrazide **1** was allowed to react with cyanoguanidine **2** and a variety of aldehydes **3a**–**3l,** namely, furfural, 1-naphthaldehyde, benzaldehyde, 4-methylbenzaldehyde, 2-methylbenzaldehyde, 3-nitrobenzaldehyde, 4-*N*, *N*-dimethylbenzaldehyde, 3-hydroxybenzaldehyde, cinnamaldehyde, acetaldehyde, 4-chloro benzaldehyde, and 3,4-dihydroxy benzaldehyde. The reaction was carried out in a one-pot, three-component system in ethanol in the presence of a few drops of conc. HCl. With stirring under reflux for 3 h, the target compounds **4**–**15** were precipitated hot and separated by filtration (Scheme 1).

**Scheme 2:**
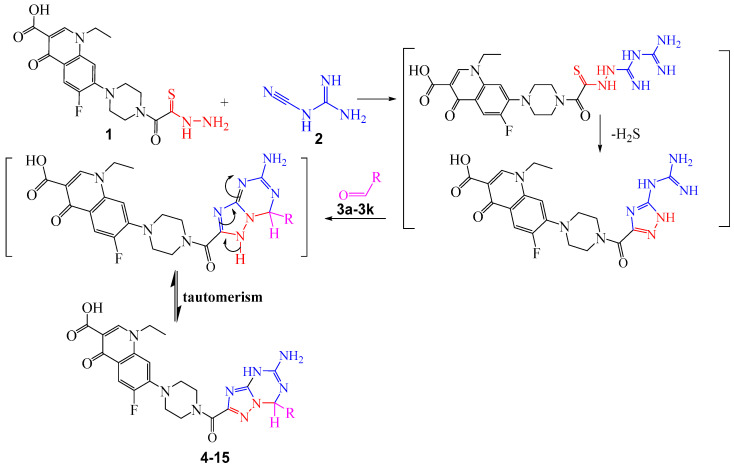
Reaction mechanism for 5-amino-7-(substituted)-N-(4-sulfamoylphenyl)-4,7-dihydro-[1,2,4]triazolo[1,5-a][1,3,5]triazine-2-carboxamide derivatives (**4**–**13**).

The reaction mechanism was assumed via a nucleophilic attack of the amino group of thiocarbohydrazide **1** at the cyano group of cyano guanidine to afford a biguanidine intermediate. This was followed by a nucleophilic attack of the NH of biguanidine at the carbon of thiocarbonyl (C=S) and the elimination of H_2_S (which was detected by paper dampened with lead acetate) to give the 5-guanidine derivative as the intermediate. In addition, the nucleophilic attack of the NH of the triazole ring and the attack of the NH of guanidine at the carbonyl group of ketone with tautomerism afford the desired triazolotriazine compounds with elimination of water (Scheme 2).

1,3,4-Triazolo[1,3,5]triazine derivatives **4**–**15** were confirmed from their physical and spectral data. The FT-IR spectrum showed the presence of an NH and NH_2_ stretching band at 3395–3170 cm^−1^, and the aromatic system was represented by a C=H stretching band at 3070–3036 cm^−1^. Also, the FT-IR spectrum showed the absence of the C=S stretching band at 1280 cm^−1^, the absence of a C-S-C stretching band at 748 cm^−1^, and the absence of C=S in CNMR spectra, providing a clear indication that the reaction had proceeded toward the formation of the triazolotriazine system. In the ^1^H NMR spectra, the signals were at 7.78–7.02 (m, CH-aromatic), 7.23–6.39 (s, 2H, NH_2,_ exchangeable by D_2_O), 5.54–5.37 (s, 1H, NH-triazine), and 4.28–4.19 (s, 1H, CH-triazine). The formation of triazolotriazine compounds **4**–**15** was proved by a clear band at 79.63–71.14 ppm in the ^13^C NMR for CH-triazine.

Herein, the target compounds **4**–**15** were prepared under different conditions to identify the best method that yields the product in high yield, in a short time, and with a green, operationally simple methodology. Initially, we selected thiocarbohydrazide **1** to react with cyanoguanidine **2** and p-methyl-benzaldehyde **3d** as model substrates for the synthesis of 7-(4-(5-amino-7-(p-tolyl)-4,7-dihydro-[1,2,4]triazolo[1,5-a][1,3,5]triazine-2-carbonyl)piperazin-1-yl)-1-ethyl-6-fluoro-4-oxo-1,4-dihydroquinoline-3-carbaldehyde (**7**) in a one-pot method to optimize the reaction conditions including the effect of type of acidic catalysis, solvent, temperature, time and yield. It is noted that the reaction rate increased with temperature; however, we observed no reaction at room temperature in the presence of AlCl_3_ (Table 1, **entry 1**). The yield of compound **10** was slightly increased to 43% upon employing a different type of acid catalyst at the same temperature and with the same solvent (Table 1, entries 2–4); however, the yield increased to 44% with a shorter reaction time (Table 1, **entry 5**). We also evaluated the effect of solvent types on product yield over the same reaction time, where acid catalysis, such as HCl, significantly improved the yield of **10** across different solvents (Table 1, **entries 6**–**8**). In line with this, dioxane gave a higher efficiency (60%) than acetonitrile (MeCN) (50%) at the same HCl concentration (1 equiv). To further prepare triazolotriazine **10** in high yield (90%), we refluxed the reaction mixture for a reduced time (Table 1, **entry 9**). Remarkably, a 91% yield of compound **10** was obtained using 1 equiv of HCl under reflux for 3 h (Table 1, entry 10). However, the reaction yield declined to 86% with an increased amount of HCl (2 equiv) at the same reaction time (Table 1, **entry 11**).

The one-pot synthetic strategy produced compound **10** in line with the principles of green chemistry. The ^1^H NMR, ^13^C NMR, and elemental analysis data of the target compounds are in good agreement with the reported data.

### 2.2. Cytotoxicity on Human Skin Carcinoma A 431

**Figure 1:**
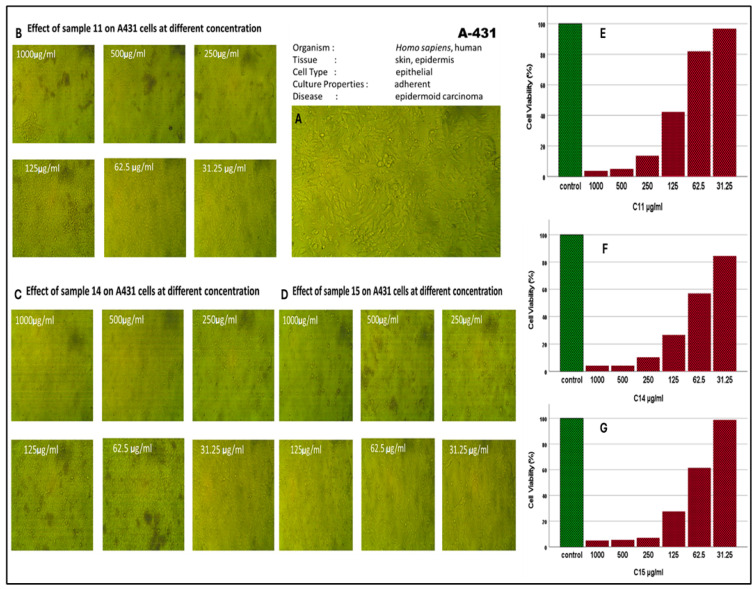
Photomicrographs showing the cytotoxic effect of the tested compounds on A 431 human skin carcinoma cells after 24 h of treatment. (**A**) Untreated control cells; (**B**) cells treated with compound **11**; (**C**) compound **14**; and (**D**) compound **15**. Panels (**E**), (**F**), and (**G**) represent the corresponding cell viability percentages for compounds **11**, **14**, and **15**, respectively, as determined by the MTT assay. Data are presented as mean ± SD from three independent experiments performed in triplicate.

**Figure 2:**
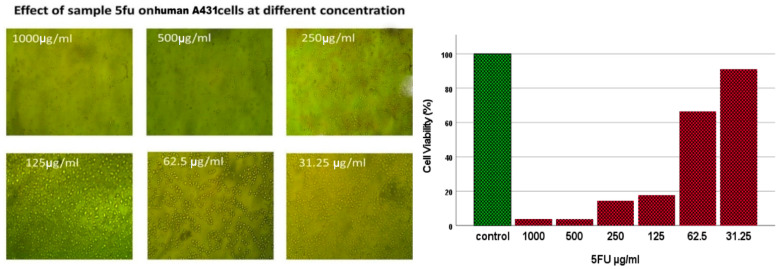
Photomicrographs showing the cytotoxic effect of 5-FU as a reference drug on A 431 human skin carcinoma cells after 24 h of treatment and corresponding cell viability percentages for it, as determined by the MTT assay. Data are presented as mean ± SD from three independent experiments performed in triplicate.

The cytotoxic effects of the target compounds **4**–**15** and the standard drug 5-fluorouracil (5-FU) were evaluated against A 431 human skin carcinoma cells using the MTT assay. The cell viability percentages and IC_50_ values were calculated to assess the potency of each compound. The standard drug 5-FU showed strong cytotoxicity with an IC_50_ value of 83.7 µg/mL, achieving 96.21% toxicity at the highest concentration (1000 µg/mL). Among the tested compounds, compound **14** demonstrated the highest potency, with an IC_50_ of 76.7 µg/mL, surpassing the efficacy of 5-FU. Compound **11** also exhibited strong activity, with an IC_50_ of 112.9 µg/mL and 96.35% toxicity at 1000 µg/mL. Similarly, compound **15** showed efficacy comparable to that of 5-FU, with an IC_50_ of 83.6 µg/mL (Figure 1 and Figure 2). IC_50_ values are listed in Table 2. In contrast, compounds such as compound **8** (IC_50_ = 208.51 µg/mL) and compound **12** (IC_50_ = 198.45 µg/mL) showed weaker cytotoxic effects. Most compounds demonstrated dose-dependent cytotoxicity, as evidenced by the gradual decrease in cell viability with increasing concentrations.

These findings highlight compounds **14**, **15**, and **11** as the most promising candidates for further development as anticancer agents targeting skin carcinoma cells. So, the morphology of their cancer cells was also found to be notably distorted in the cell line at higher concentrations of the tested compounds after 24 h of treatment (Figure 1 and Figure 2).

### 2.3. Cytotoxicity on Normal Skin Cell Line BJ

The cytotoxic effects of the most active anticancer compounds, **14** and **15**, were evaluated in comparison with camptothecin on the normal skin cell line BJ to assess their selectivity toward cancer cells. As shown in Table 3, both compounds **14** and **15** demonstrated a markedly safer profile than camptothecin with IC_50_ values of 61.8, 43.8, and 27.8, respectively. Compound **14** exhibited an IC_50_ value more than twice that of camptothecin, indicating greater selectivity toward cancer cells. This superior safety index of compound **14** justified its selection for further mechanistic studies, including Topo I/II inhibition, cell cycle analysis, and apoptosis assays.

### 2.4. Topoisomerase I/II Inhibition Assay

Compound **14**, the most potent and selective among the synthesized series, was screened for inhibitory activity against Topo I and II, compared with the reference drugs camptothecin and doxorubicin, respectively [4]. Compound **14** showed moderate inhibition of Topo I with an IC_50_ of 17.5 ± 0.59 µM, whereas camptothecin exhibited stronger activity (IC_50_ = 2.485 ± 0.08 µM). Similarly, compound **14** showed weaker inhibition of Topo II (IC_50_ = 17.3 ± 1.02 µM) relative to doxorubicin (IC_50_ = 1.14 ± 0.07 µM). These findings suggest that the potent anticancer activity of compound **14** is not solely dependent on Topo I/II inhibition, indicating the involvement of additional mechanisms, such as induction of apoptosis and cell cycle arrest (Table 4).

### 2.5. Cell Cycle Analysis

**Figure 3:**
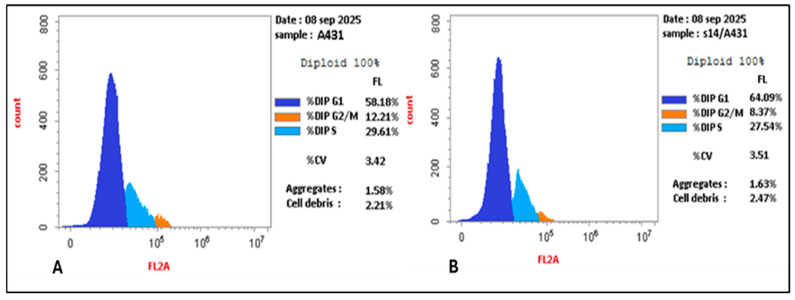
Effect of compound **14** (IC_50_ = 76.7 µg) on the distribution of A 431 skin cancer cells across different phases of the cell cycle (**B**) compared with the untreated negative control (**A**).

The impact of compound **14** on the cell cycle progression of A 431 skin cancer cells was assessed via flow cytometry according to the reported protocols [4]. The current study performed cell cycle analysis on an asynchronous population of A 431 cells without prior synchronization. The baseline cell cycle distribution was determined using the DMSO-treated control group, which represents the inherent cell cycle profile under the same experimental conditions. Despite the absence of cell synchronization (e.g., by serum starvation or chemical arrest), all experimental groups, including those treated with compound **14** and DMSO control cells—originated from the same initial cell population and were sustained under uniform culture conditions. This methodology established a uniform baseline among groups, facilitating a rigorous and reliable comparative examination of treatment-induced impacts on cell cycle progression. Treatment at the IC_50_ concentration of compound **14** induced a significant G0/G1 phase arrest, with 64.09% of cells accumulating in this phase, compared with 58.18% in the DMSO control group. A concomitant reduction was noted in the S phase (27.54% vs. 29.61%) and G2/M phase (8.37% vs. 12.21%). The results demonstrate that compound **14** efficiently obstructs the G1-to-S transition, consequently impeding DNA synthesis and mitotic progression (Table 5 and Figure 3).

### 2.6. Apoptosis Assay

**Figure 4:**
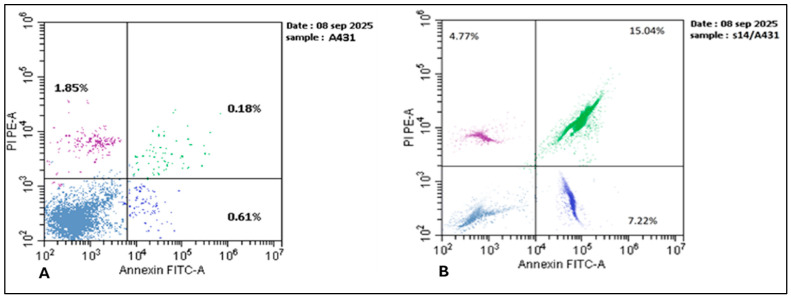
Flow cytometry dot plots of A 431 skin cancer cells after Annexin V-FITC/PI staining. (**A**) Untreated control cells; (**B**) cells treated with compound **14** at its IC_50_ concentration (76.7 µg). Quadrants represent viable cells (LL), early apoptotic cells (LR), necrotic cells (UL), and late apoptotic cells (UR).

The pro-apoptotic activity of compound **14** was assessed in A 431 cells using Annexin V-FITC/PI staining as per the reported protocols [4]. Annexin V binds to phosphatidylserine residues that become exposed to the outer leaflet of the plasma membrane during early apoptosis, while propidium iodide (PI) enters cells with compromised membranes, indicating late apoptosis or necrosis. Thus, Annexin V-positive/PI-negative cells represent early apoptotic cells, whereas Annexin V-positive/PI-positive cells indicate late apoptotic or necrotic cells. Treatment with the IC_50_ concentration of compound **14** induced significant levels of early apoptosis (7.22%), late apoptosis (15.04%), and necrosis (4.77%), whereas the untreated group showed negligible apoptotic activity. These findings confirm that the anticancer effect of compound **14** is mediated, at least in part, through induction of apoptosis and necrosis, supporting its potential as an effective anticancer candidate (Table 6 and Figure 4).

### 2.7. Docking Studies

Docking simulations of compound **14** were conducted in the DNA cleavage complexes of human Topo I (PDB ID: 1K4T) and human Topo IIβ (PDB ID: 7YQ8), utilizing camptothecin and doxorubicin as reference ligands, respectively. The docking methodology was confirmed by redocking the co-crystallized ligands, which replicated the experimental binding orientations and affirmed the trustworthiness of the employed workflow for pose analysis. Ternary enzyme–DNA complexes are particularly suitable for topoisomerase poisons, as their efficacy relies on the stabilization of the cleavage complex by concurrent interactions with both the protein and DNA bases at the cleavage site.

The computed docking scores demonstrated advantageous binding of the target compound **14** within the two topoisomerase cleavage complexes. In Topo I (1K4T), compound **14** yielded a docking score of −7.9 kcal/mol in comparison to the reference camptothecin with a docking score of −8.5 kcal/mol (Table 7), while in Topo IIβ (7YQ8), the corresponding value was −9.2 kcal/mol in comparison to the reference doxorubicin with a docking score of −9.3 kcal/mol (Table 8). These values facilitate the effective accommodation of compound **14** in the target binding areas; nonetheless, they should be regarded as relative markers of pose favourability within the examined series rather than direct quantitative predictions of experimental potency. The docking technique was primarily used to elucidate residue- and nucleotide-level interactions that may explain the observed biological activity.

**Figure 5:**
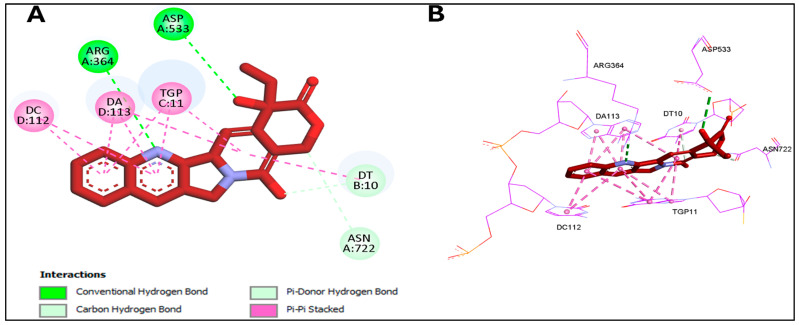
(**A**) Two-dimensional interactions (**B**) and 3D interactions of camptothecin in the Topo I active site (PDB: 1K4T).

**Figure 6:**
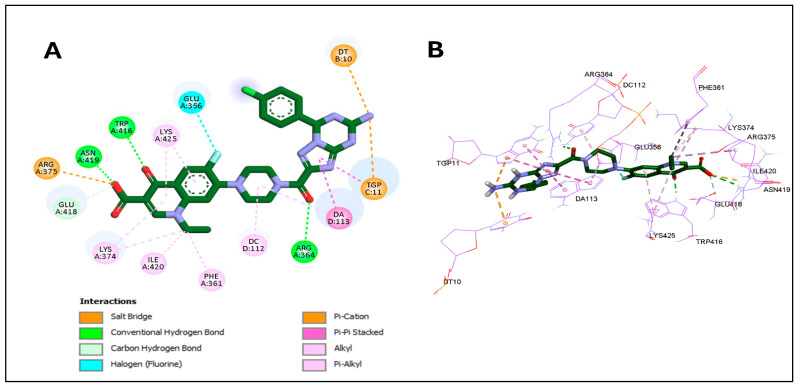
(**A**) Two-dimensional interaction in the Topo I active site (PDB: 1K4T) for compound **14**; (**B**) 3D binding interactions of compound **14**.

In the Topo I–DNA cleavage complex (Figure 5 and Figure 6), compound **14** exhibited binding conformations that align with cleavage-complex stability via a combination of polar, electrostatic, hydrophobic, and π-interactions. Compound **14** carboxylate oxygen established a salt bridge with ASN 419, while the quinolone 4-oxo carbonyl oxygen made a hydrogen bond with TRP 416, and the carbonyl linker formed supplementary hydrogen connections with ARG 364. The conjugated aromatic system formed π–π stacking interactions with DNA bases (including DA113, DC112, and DT10), which mechanistically aligns with topoisomerase poisoning behaviour, as these interactions may stabilize the trapped cleavage complex and inhibit DNA religation.

**Figure 7:**
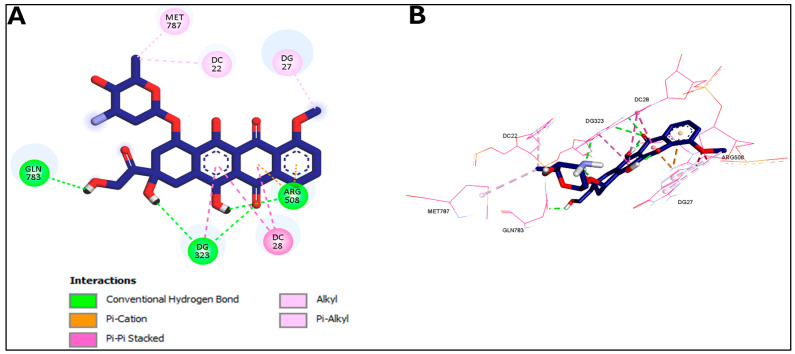
(**A**) Two-dimensional interactions; (**B**) 3D interactions of doxorubicin in Topo IIβ active site (PDB: 7YQ8).

**Figure 8:**
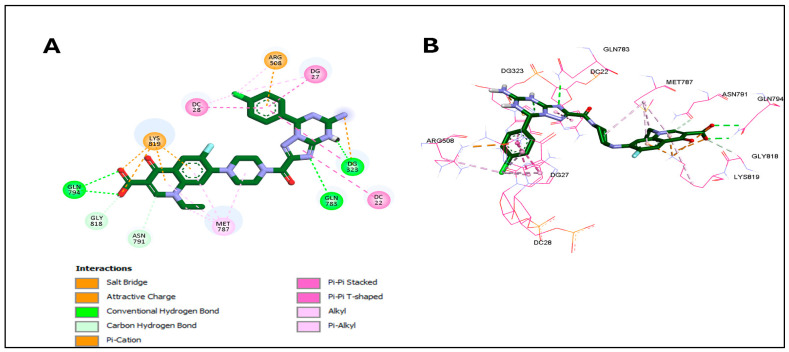
(**A**) Two-dimensional interactions in Topo IIβ active site (PDB: 7YQ8); (**B**) 3D binding interactions of the target compound **14**.

Furthermore, compound **14** maintained the identical critical Topo IIβ anchoring connections, which correlate with its enhanced biological efficacy (Figure 7 and Figure 8). The quinolone carboxylate oxygen exhibited a strong interaction with LYS 819, establishing two hydrogen bonds with GLN 794. At the same time, the triazole nitrogen produced a hydrogen link with GLN 783, and the substituted amino group of the triazine ring formed a hydrogen bond with DG 323. The inserted aromatic moiety sustained π-π and hydrophobic interactions with DNA bases, specifically DG 27, DC 22, and DC 28. The heightened contact density noted for compound **14**, especially at the DNA interface and within amino acid residues ARG 508, GLN 794, LYS819, and MET787, offers a persuasive structural justification for its inhibition of Topo I and Topo IIβ in the cellular assay.

The binding scores of the target compounds are slightly less than those of the reference drugs on both Topo I/II, which goes ahead with the results of the Topo I/II assay and confirms that the activity of the target compounds is related to multiple mechanisms such as cell cycle arrest and induction of apoptosis in addition to Topo I/II inhibition.

## 3. Discussion

This work examined the anticancer efficacy of freshly synthesized N^4^-(5-Amino-7-substituted-triazolotriazinepiperazin-1-yl) norfloxacin analogues. The cytotoxicity data from A 431 skin cancer cells, compared with the normal BJ skin cell line, indicated that both compounds **14** and **15** exhibit significant anticancer efficacy while maintaining a favourable safety profile in normal cells. The elevated IC_50_ values in BJ cells indicate reduced toxicity to healthy tissues, suggesting that these compounds exhibit favourable selectivity for malignant cells, a crucial characteristic in contemporary anticancer drug development. Notably, compound **14** consistently demonstrated superior action compared to compound **15**, as seen by its lower IC_50_ values and greater inhibitory efficacy across all evaluated assays. This increased efficacy indicates that compound **14** possesses a greater capacity to inhibit cancer cell proliferation while exerting little detrimental effects on normal cells. Selectivity is an essential attribute, as numerous traditional medicines, including classical topoisomerase inhibitors and genetically targeted drugs, frequently harm normal tissues, resulting in significant adverse consequences. Recent research emphasizes that anticancer medicines that specifically target tumour cells while preserving healthy cells signify significant progress in therapeutic development. Numerous natural and synthetic substances have demonstrated selectivity, underscoring the importance of this characteristic in advancing safer anticancer pharmaceuticals [36,37,38].

Compound **14** was assessed for its inhibitory effect on Topo I/II, revealing that it inhibits both enzymes but with reduced efficacy compared with the conventional reference inhibitors (camptothecin for Topo I and doxorubicin for Topo II). The data suggests that the anticancer efficacy of compound **14** is not only attributable to topoisomerase inhibition but also probably augmented by additional mechanisms, including induction of apoptosis and cell cycle arrest.

This multi-mechanistic drug action is beneficial, as it induces DNA damage or disrupts cell cycle progression via one pathway while concurrently initiating programmed cell death through another, thereby significantly enhancing anticancer efficacy and potentially diminishing the risk of resistance, a frequent problem encountered with single-mechanism topoisomerase inhibitors. Prior research has demonstrated that topoisomerase inhibitors can induce apoptosis via multiple molecular pathways, including caspase activation, mitochondrial dysfunction, and DNA damage recognition [37,39].

It is crucial to acknowledge that certain topoisomerase inhibitors may induce adverse effects, including increased reactive oxygen species (ROS), which may subsequently promote cancer cell migration. One study indicated that topoisomerase inhibitors activate the JAK2-STAT1–CXCL1 signalling pathway via ROS production, thereby increasing cell motility [40]. Compound **14** induced a significant increase in A 431 cancer cells in the G0/G1 phase, reaching approximately 64% compared with the control group. The G1-phase arrest holds significant therapeutic value, as it prevents cells from entering the DNA synthesis (S) phase, thereby curtailing cellular growth.

Clinically, targeting cell-cycle checkpoints such as G1/S is recognized as an effective anticancer strategy due to their frequent dysregulation in cancer cells. This deficiency can be therapeutically exploited, rendering malignant cells more susceptible to therapy, while normal cells maintain superior checkpoint control and hence exhibit a distinct response. This approach is well supported by basic clinical research, including a paper that details how exploiting checkpoint defects is a fundamental principle in cancer therapy [41].

Moreover, numerous recent investigations indicate that G0/G1 arrest triggered by new drugs facilitates selective apoptosis in cancer cells while preserving normal cells [36,38]. In accordance with these findings, Annexin V-FITC/PI labelling demonstrated that compound **14** produces substantial early and late apoptosis, alongside a reduced fraction of necrotic cells. Apoptosis is the favoured mechanism of cancer cell death, as it prevents inflammation and facilitates the regulated removal of dangerous cells. Topoisomerase inhibitors induce apoptosis by activating DNA damage pathways involving ATM/ATR, Chk2, and mitochondrial signalling, consistent with the reported apoptotic profile [38].

This investigation reveals that compound **14** exhibits enhanced anticancer efficacy relative to compound **15**, as evidenced by its lower IC_50_ against A 431 cells and its greater capacity to induce apoptosis and cell-cycle arrest at the G0/G1 phase. Both drugs, however, exhibited a favourable safety profile in the normal BJ cell line, demonstrating significant selectivity, which is crucial for advancing promising anticancer prospects.

Compound **14** had modest inhibitory action against Topo I and II; nonetheless, its anticancer efficacy exceeded expectations based solely on topoisomerase inhibition. This indicates that other mechanisms, such as apoptosis induction and disruption of cell cycle progression, play a substantial role in its cytotoxic effects. This multi-pathway approach is beneficial because it reduces the likelihood of resistance emergence and improves overall treatment effectiveness. Notwithstanding these advantages, some factors persist. The identified necrotic response, while facilitating cell death, may induce inflammation in vivo and necessitate additional assessment. Similarly, the potential for reactive oxygen species formation, noted for certain topoisomerase inhibitors in prior research, must be investigated to confirm that compound **14** does not activate unwanted pro-migratory pathways. Furthermore, the present findings are confined to in vitro assessments using the A 431 model and require validation across several cancer cell lines and animal models.

Subsequent research should encompass in vivo efficacy and toxicity assessments, comprehensive molecular characterization of apoptotic and DNA damage pathways, and structural optimization to enhance potency while mitigating potential adverse effects. The results indicate that compound **14** is a more effective and promising anticancer candidate than compound **15**, and a robust mechanistic basis for further preclinical development supports this.

Regarding the Structure–Activity Relationship (SAR) of the target compounds (**4**–**15**), it is evident that the variations in the R group of the aldehyde moiety (methyl, phenyl, naphthyl, furyl) do not significantly affect the activity against the tested skin cancer cell line, with IC_50_ values ranging from 118 to 154 µM. Furthermore, derivative **8**, featuring the 2-methylphenyl moiety, exhibited a notable reduction in activity relative to compound **7**, which contains the 4-methylphenyl moiety. This observation may suggest that the steric effect influences binding to the target enzyme. Furthermore, the electrical influence of the substituted phenyl group exhibited minimal impact on the anticancer efficacy of the target compounds, except for p-chlorophenyl in compound **14**, which demonstrated the highest potency with an IC_50_ value of 76.7 µM.

## 4. Materials and Methods

### 4.1. Chemistry

All prepared target compounds were identified by melting point measurements using the Fisher–John mechanical technique, along with spectroscopic techniques and mass analysis. Instrumentation and Chemicals: Chemicals and solvents were purchased from Sigma-Aldrich, Darmstadt, Germany. The IR (ν cm^−1^) spectra of the prepared compounds were analyzed using the KBr method, and ^1^H NMR (δ ppm) and ^13^C NMR spectra were recorded on the spectrometer model Bruker ADVANCE 400 MHz (Bruker Corporation, Billerica, MA, USA).

#### Synthesis of the Target Compounds **4**–**15**

General Procedure:

To a solution of previously reported thiohydrazide derivative [42] (1 mmol) in ethanol (20 mL), (1 mmol) of cyanoguanidine, (1 mmol) of aldehyde derivatives, and 0.5 mL of conc. HCl were added, and the reaction mixture was refluxed with stirring for about 8 h. The reaction mixture was cooled, and the precipitated solid was collected by filtration, washed with ethanol, and dried to afford the final pure compounds.

7-(4-(5-Amino-7-(furan-2-yl)-4,7-dihydro-[1,2,4]triazolo[1,5-a][1,3,5]triazine-2-carbonyl)piperazin-1-yl)-1-ethyl-6-fluoro-4-oxo-1,4-dihydroquinoline-3-carboxylic acid (**4**).

Brown precipitate; Yield (79%, 0.434 g); mp: 267–268 °C; ^1^H NMR (δ ppm) 15.21 (s, 1H, COOH), 9.81 (s, 1H, H-2 of quinolone), 8.90 (d, *J_H-F_* = 13.6 Hz, 1H, H-5 of quinolone), 7.94 (d, *J_H-F_* = 7.6 Hz, 1H, H-8 of quinolone), 7.40–7.09 (m, 3H, Ar), 6.87 (s, 2H, NH_2_), 5.37 (s, 1H, NH), 4.59 (q, *J_H-H_* = 7 Hz, 2H, -CH_2_-CH_3_), 4.28 (br, 1H, CH), 3.33–3.30 (m, 8H, piperazine), 1.43 (t, *J_H-H_* = 7 Hz, 3H, -CH_2_CH_3_); ^13^C NMR (DMSO-*d*_6_) δ ppm: 176.25, 166.78, 166.07, 153.39, 152.57, 152.41, 150.62, 149.56, 147.47, 142.14, 139.37, 120.06, 112.57, 110.21, 109.08, 106.60, 106.01, 79.63, 53.55, 51.88, 49.29, 14.65; C.F: C_25_H_24_FN_9_O_5_; M.W: 549.52; Anal. Calcd: C, 54.64; H, 4.40; N, 22.94; Found: C, 54.60; H, 4.43; N, 22.89.

7-(4-(5-Amino-7-(naphthalen-1-yl)-4,7-dihydro-[1,2,4]triazolo[1,5-a][1,3,5]triazine-2-carbonyl)piperazin-1-yl)-1-ethyl-6-fluoro-4-oxo-1,4-dihydroquinoline-3-carboxylic acid (**5**)

Pale yellow precipitate; Yield (85%, 0.517 g); mp: 270–272 °C; ^1^H NMR (δ ppm) 15.21 (s, 1H, COOH), 8.92 (s, 1H, H-2 of quinolone), 8.52 (d, *J_H-F_* = 13.6 Hz, 1H, H-5 of quinolone), 8.28–7.28 (m, 7H, Ar-H), 7.08 (d, *J_H-F_* = 7.5 Hz, 1H, H-8 of quinolone), 6.75 (s, 2H, NH_2_), 5.45 (s, 1H, NH), 4.60 (q, *J_H-H_* = 7 Hz, 2H, -CH_2_-CH_2_CH_3_), 4.28 (br, 1H, CH), 3.94–3.33 (m, 8H, piperazine), 1.44 (t, *J_H-H_* = 7 Hz, 3H, -CH_2_CH_3_); ^13^C NMR (DMSO-*d*_6_) δ ppm: 176.26, 166.72, 166.37, 155.40, 154.44, 153.66, 152.65, 150.24, 149.53, 147.38, 143.52, 139.34, 134.44, 133.79, 132.65, 128.21, 125.56, 125.29, 120.04, 112.58, 111.33, 109.46, 106.31, 76.59, 53.66, 51.20, 49.59, 14.85; C.F: C_31_H_28_FN_9_O_4_; M.W: 609.62; Anal. Calcd: C, 61.08; H, 4.63; N, 20.68; Found: C, 61.15; H, 4.60; N, 20.64.

7-(4-(5-Amino-7-phenyl-4,7-dihydro-[1,2,4]triazolo[1,5-a][1,3,5]triazine-2-carbonyl-)piperazin-1-yl)-1-ethyl-6-fluoro-4-oxo-1,4-dihydroquinoline-3-carboxylic acid. (**6**)

Yellow precipitate; Yield (90%, 0.504 g); mp: 258–260 °C; ^1^H NMR (δ ppm): 15.21 (s, 1H, COOH), 8.93 (s, 1H, H-2 of quinolone), 7.89 (d, *J_H-F_* = 13.6 Hz, 1H, H-5 of quinolone), 7.61–7.11 (m, 5H, Ar-H), 6.75 (d, *J_H-F_* = 7.5 Hz, 1H, H-8 of quinolone), 6.05 (s, 2H, NH_2_), 5.42 (s, 1H, NH), 4.61 (q, *J_H-H_* = 7 Hz, 2H, -CH_2_-CH_3_), 4.19 (br, 1H, CH), 3.76–3.33 (m, 8H, piperazine), 1.43 (t, *J_H-H_* = 7 Hz, 3H, -CH_3_); ^13^C NMR (DMSO-*d*_6_) δ ppm: 176.26, 166.72, 166.03, 153.03, 152.09, 150.92, 149.83, 147.75, 143.59, 139.35, 137.91, 129.81, 128.68, 125.26, 120.39, 112.36, 109.13, 106.67, 106.35, 78.99, 53.64, 51.21, 49.03, 14.60; C.F: C_27_H_26_FN_9_O_4_; M.W: 559.56; Anal. Calcd: C, 57.96; H, 4.68; N, 22.53; Found: C, 57.98; H, 4.66; N, 22.55.

7-(4-(5-Amino-7-(p-tolyl)-4,7-dihydro-[1,2,4]triazolo[1,5-a][1,3,5]triazine-2-carbonyl-)piperazin-1-yl)-1-ethyl-6-fluoro-4-oxo-1,4-dihydroquinoline-3-carboxylic acid. (**7**)

Yellow precipitate; Yield (91%, 0.521 g); mp: 280–282 °C; ^1^H NMR (δ ppm): 15.21 (s, 1H, COOH), 8.93 (s, 1H, H-2 of quinolone), 7.87 (d, *J_H-F_* = 13.6 Hz, 1H, H-5 of quinolone), 7.45–7.16 (m, 4H, Ar), 6.75 (s, 2H, NH_2_), 6.34 (d, *J_H-F_* = 7.6 Hz, 1H, H-8 of quinolone), 5.51 (s, 1H, NH), 4.66 (q, *J_H-H_* = 7 Hz, 2H, -CH_2_-CH_3_), 4.28 (br, 1H, CH), 3.92–3.41 (m, 8H, piperazine), 2.28 (s, 3H, CH_3_), 1.45 (t, *J_H-H_* = 7 Hz, 3H, -CH_2_CH_3_); ^13^C NMR (DMSO-*d*_6_) δ ppm: 176.28, 166.78, 166.45, 153.28, 152.65, 150.95, 149.91, 147.80, 143.48, 139.33, 135.08, 134.07, 128.76, 128.46, 120.00, 112.61, 109.17, 106.38, 78.92, 53.59, 51.14, 49.02, 21.30, 14.63; C.F: C_28_H_28_FN_9_O_4_; M.W: 573.59; Anal. Calcd: C, 58.63; H, 4.92; N, 21.98; Found: C, 58.61; H, 4.94; N, 21.96.

7-(4-(5-Amino-7-(o-tolyl)-4,7-dihydro-[1,2,4]triazolo[1,5-a][1,3,5]triazine-2-carbonyl-)piperazin-1-yl)-1-ethyl-6-fluoro-4-oxo-1,4-dihydroquinoline-3-carboxylic acid (**8**)

Yellow precipitate; Yield (75%, 0.420 g); mp: 268–270 °C; ^1^H NMR (δ ppm): 15.21 (s, 1H, COOH), 8.93 (s, 1H, H-2 of quinolone), 7.89 (d, *J_H-F_* = 13.6 Hz, 1H, H-5 of quinolone), 7.45–7.16 (m, 4H, Ar), 6.75 (s, 2H, NH_2_), 6.34 (d, *J_H-F_* = 7.5 Hz, 1H, H-8 of quinolone), 5.51 (s, 1H, NH), 4.66 (q, *J_H-H_* = 7 Hz, 2H, -CH_2_-CH_3_), 4.28 (br, 1H, CH), 3.92–3.57 (m, 8H, piperazine), 2.28 (3H, CH_3_), 1.43 (t, *J_HH_* = 7Hz, 3H, CH_2_CH_3_); ^13^C NMR (DMSO-*d*_6_) δ ppm: 176.26, 166.72, 166.03, 153.03, 152.09, 150.92, 149.83, 147.75, 143.59, 139.35, 137.91, 129.81, 128.68, 125.26, 120.39, 112.36, 109.13, 106.73, 106.35, 78.99, 53.64, 51.21, 49.03, 14.60; C.F: C_27_H_26_FN_9_O_4_; M.W: 559.56; Anal. Calcd: C, 57.96; H, 4.68; N, 22.53; Found: C, 57.98; H, 4.66; N, 22.55.

7-(4-(5-Amino-7-(3-nitrophenyl)-4,7-dihydro-[1,2,4]triazolo[1,5-a][1,3,5]triazine-2-carbonyl)piperazin-1-yl)-1-ethyl-6-fluoro-4-oxo-1,4-dihydroquinoline-3-carboxylic acid. (**9**)

Yellow precipitate; Yield (77%, 0.465 g); mp: 228–230 °C; ^1^H NMR (δ ppm): 15.21 (s, 1H, COOH), 8.94 (s, 1H, H-2 of quinolone), 8.69 (s, H, Ar-H), 7.96 (d, *J_H-F_* = 13.6 Hz, 1H, H-5 of quinolone), 7.38–7.07 (m, 4H, 3Ar-H+ H-8 of quinolone), 6.69 (s, 2H, NH_2_), 5.54 (s, 1H, NH), 4.61 (q, *J_H-H_* = 7.6 Hz, 2H, -CH_2_-CH_3_), 4.22 (br, 1H, CH), 3.76–3.33 (m, m, 8H, piperazine), 1.43 (t, *J_H-H_* = 7 Hz, 3H, -CH_3_); ^13^C NMR (DMSO-*d*_6_) δ ppm: 176.61, 166.72, 166.49, 153.36, 152.00, 150.84, 149.51, 147.82, 147.14, 143.50, 139.02, 138.32, 135.45, 129.49, 125.27, 120.76, 120.43, 112.60, 109.14, 106.96, 77.89, 53.24, 51.51, 49.60, 14.81; C.F: C_27_H_25_FN_10_O_6_; M.W: 604.56; Anal. Calcd: C, 53.64; H, 4.17; N, 23.17; Found: C, 53.61; H, 4.12; N, 23.24.

7-(4-(5-Amino-7-(4-(dimethylamino)phenyl)-4,7-dihydro-[1,2,4]triazolo[1,5-a][1,3,5]-triazine-2-carbonyl)piperazin-1-yl)-1-ethyl-6-fluoro-4-oxo-1,4-dihydroquinoline-3-carboxylic acid (**10**)

Red precipitate; Yield (76%, 0.458 g). mp: 200–202 °C; ^1^H NMR (δ ppm): 15.21 (s, 1H, COOH), 8.94 (s, 1H, H-2 of quinolone), 7.94 (d, *J_H-F_* = 13.6 Hz, 1H, H-5 of quinolone), 7.33–7.02 (m, 4H, Ar-H), 6.80 (d, *J_H-F_* = 7.6 Hz, 1H, H-8 of quinolone), 6.45 (s, 2H, NH_2_), 5.42 (s, 1H, NH), 4.60 (q, *J_H-H_* = 7 Hz, 2H, -CH_2_-CH_3_), 4.23 (br, 1H, CH), 3.94–3.90 (m, 2H, piperazine), 3.76–3.73 (m, 2H, piperazine), 3.69–3.65 (m, 4H, piperazine), 3.05 (s, 6H, N(CH_3_)_2_). 1.44 (t, *J_H-H_* = 7 Hz, 3H, -CH_3_); ^13^C NMR (DMSO-*d*_6_) δ ppm: 176.28, 166.45, 166.03, 153.28, 152.34, 150.21, 149.50, 148.82, 146.73, 143.13, 139.71, 129.48, 127.77, 120.41, 112.61, 112.29, 109.53, 106.66, 78.92, 53.59, 51.91, 49.02, 42.18, 14.29; C.F: C_29_H_31_FN_10_O4; M.W: 602.63; Anal. Calcd: C, 57.80; H, 5.19; N, 23.24; Found: C, 57.74; H, 5.13; N, 23.19.

7-(4-(5-Amino-7-(3-hydroxyphenyl)-4,7-dihydro-[1,2,4]triazolo[1,5-a][1,3,5]triazine-2-carbonyl)piperazin-1-yl)-1-ethyl-6-fluoro-4-oxo-1,4-dihydroquinoline-3-carboxylic acid (**11**)

Pale yellow precipitate; Yield (80%, 0.460 g); mp: 248–250 °C; ^1^H NMR (δ ppm): 15.25 (s, 1H, COOH), 9.81 (s, 1H, OH), 8.94 (s, 1H, H-2 of quinolone), 7.94 (d, *J_H-F_* = 13.6 Hz, 1H, H-5 of quinolone), 7.33–7.02 (m, 4H, Ar-H), 6.66 (d, *J_H-F_* = 7.6 Hz, 1H, H-8 of quinolone), 6.39 (s, 2H, NH_2_), 5.39 (s, 1H, NH), 4.60 (q, *J_H-H_* = 7 Hz, 2H, -CH_2_-CH_3_), 4.23 (br, 1H, CH), 3.76–3.33 (m, 8H, piperazine), 1.44 (t, *J_H-H_* = 7 Hz, 3H, -CH_3_); ^13^C NMR (DMSO-*d*_6_) δ ppm: 176.42, 166.69, 166.03, 156.83, 153.35, 152.11, 150.88, 149.82, 147.10, 143.20, 139.79, 139.01, 130.58, 121.13, 120.69, 114.35, 112.89, 112.25, 109.13, 106.85, 78.19, 53.68, 51.19, 49.45, 14.68. C.F: C_27_H_26_FN_9_O_5_; M.W: 575.56; Anal. Calcd: C, 56.34; H, 4.55; F, 3.30; N, 21.90; Found: C, 56.27; H, 4.60; N, 21.84.

7-(4-(5-Amino-7-styryl-4,7-dihydro-[1,2,4]triazolo[1,5-a][1,3,5]triazine-2-carbonyl)-piperazin-1-yl)-1-ethyl-6-fluoro-4-oxo-1,4-dihydroquinoline-3-carboxylic acid. (**12**)

Yellow precipitate; Yield (75.8%, 0.442 g); mp: 260–262 °C; ^1^H NMR (δ ppm): 15.25 (s, 1H, COOH), 8.93 (s, 1H, H-2 of quinolone), 7.92 (d, *J_H-F_* = 13.6 Hz, 1H, H-5 of quinolone), 7.78–7.07 (m, 6H, Ar-H+ H-8 of quinolone), 6.66 (s, 2H, NH_2_), 6.22 (s, 2H, CH=CH), 5.42 (s, 1H, NH), 4.58 (q, *J_H-H_* = 7 Hz, 2H, -CH_2_-CH_3_), 4.28 (br, 1H, CH), 3.97–3.93 (broad, 2H, piperazine), 3.74–3.70 (broad, 2H, piperazine), 3.50–3.33 (broad, 4H, piperazine), 1.43 (t, *J_H-H_* = 7 Hz, 3H, -CH_2_CH_3_); ^13^C NMR (DMSO-*d*_6_) δ ppm: 176.87, 166.77, 166.04, 153.60, 152.99, 150.62, 149.54, 147.43, 143.92, 139.00, 136.81, 130.94, 128.83, 128.42, 127.49, 121.71, 120.79, 112.06, 109.11, 106.65, 77.47, 53.63, 51.19, 49.64, 14.67; C.F: C_29_H_28_FN_9_O_4_; M.W: 585.60; Anal. Calcd: C, 59.48; H, 4.82; N, 21.53; Found: C, 59.39; H, 4.79; N, 21.47.

7-(4-(5-Amino-7-methyl-4,7-dihydro-[1,2,4]triazolo[1,5-a][1,3,5]triazine-2-carbonyl)-piperazin-1-yl)-1-ethyl-6-fluoro-4-oxo-1,4-dihydroquinoline-3-carboxylic acid (**13**)

Yellow precipitate; Yield (77%, 0.383 g); mp: 258–260 °C; ^1^H NMR (δ ppm): 15.21 (s, 1H, COOH), 8.93 (s, 1H, H-2 of quinolone), 7.95 (d, *J_H-F_* = 13.6 Hz, 1H, H-5 of quinolone), 7.23 (s, 2H, NH_2_), 6.99 (d, *J_H-F_* = 7.6 Hz, 1H, H-8 of quinolone), 5.39 (s, 1H, NH), 4.60 (q, *J_H-H_* = 7 Hz, 2H, -CH_2_-CH_3_), 4.22 (br, 1H, CH), 3.76–3.88 (m, 8H, piperazine), 1.83 (s, 3H, CH_3_), 1.43 (t, *J_HH_* =7Hz, 3H, CH_2_CH_3_); ^13^C NMR (DMSO-*d*_6_) δ ppm: 176.60, 166.73, 166.37, 153.38, 152.02, 150.20, 149.86, 147.44, 143.54, 139.71, 120.44, 112.30, 109.87, 106.64, 71.14, 53.18, 51.27, 49.05, 21.25, 14.60; C.F: C_22_H_24_FN_9_O_4_; M.W: 497.49; Anal. Calcd: C, 53.11; H, 4.86; N, 25.34; Found: C, 53.13; H, 4.84; N, 25.36.

7-(4-(5-Amino-7-(4-chlorophenyl)-4,7-dihydro-[1,2,4]triazolo[1,5-a][1,3,5]triazine-2-carbonyl)piperazin-1-yl)-1-ethyl-6-fluoro-4-oxo-1,4-dihydroquinoline-3-carboxylic acid (**14**)

Pale yellow precipitate; Yield (75%, 0.446 g). mp: 222–225 °C; ^1^H NMR (δ ppm): 15.21 (s, 1H, COOH), 8.94 (s, 1H, H-2 of quinolone), 7.95 (d, *J_H-F_* = 13.6 Hz, 1H, H-5 of quinolone), 7.37–7.06 (m, 4H, Ar-H), 6.72 (d, *J_H-F_* = 7.6 Hz, 1H, H-8 of quinolone), 6.48 (s, 2H, NH_2_), 5.37 (s, 1H, NH), 4.63 (q, *J_H-H_* = 7 Hz, 2H, -CH_2_-CH_3_), 4.25 (br, 1H, CH), 3.76–3.33 (m, 8H, piperazine), 1.43 (t, *J_H-H_* = 7 Hz, 3H, -CH_2_CH_3_); ^13^C NMR (DMSO-*d*_6_) δ ppm: 176.20, 166.36, 166.04, 153.39, 152.64, 150.65, 149.52, 147.05, 143.17, 139.00, 135.47, 131.62, 130.88, 128.40, 120.39, 112.56, 109.43, 106.91, 78.94, 53.94, 51.89, 49.46, 14.87; C.F: C_27_H_25_ClFN_9_O_4_; M.W: 594.00; Anal. Calcd: C, 54.60; H, 4.24; N, 21.22; Found: C, 54.52; H, 4.16; N, 21.16.

7-(4-(5-Amino-7-(3,4-dihydroxyphenyl)-4,7-dihydro-[1,2,4]triazolo[1,5-a][1,3,5]triazine-2-carbonyl)piperazin-1-yl)-1-ethyl-6-fluoro-4-oxo-1,4-dihydroquinoline-3-carboxylic acid (**15**)

Pale yellow precipitate; Yield (91%, 0.502 g); mp: 258–260 ^°C^; ^1^H NMR (δ ppm): 15.21 (s, 1H, COOH), 9.69 (s, 1H, COOH), 8.92 (s, 1H, H-2 of quinolone), 7.91 (d, *J_H-F_* = 13.6 Hz, 1H, H-5 of quinolone), 7.43–7.12 (m, 3H, Ar-H), 6.94 (d, *J_H-F_* = 7.6 Hz, 1H, H-8 of quinolone), 6.60 (s, 2H, NH_2_), 5.39 (s, 1H, NH), 4.59 (q, *J_H-H_* = 7 Hz, 2H, -CH_2_-CH_3_), 4.23 (br, 1H, CH), 3.94–3.66 (m, 8H, piperazine), 1.44 (t, *J_H-H_* = 7 Hz, 3H, -CH_2_CH_3_); ^13^C NMR (DMSO-*d*_6_) δ ppm: 176.28, 166.72, 166.49, 153.73, 152.62, 150.58, 149.51, 147.14, 145.66, 144.26, 143.99, 139.37, 131.59, 123.93, 120.43, 116.60, 115.09, 112.94, 109.82, 106.96, 78.60, 53.59, 51.51, 49.60, 14.81.C.F: C_27_H_26_FN_9_O_6_; M.W: 591.56; Anal. Calcd: C, 54.82; H, 4.43; N, 21.31; Found: C, 54.68; H, 4.39; N, 21.26.

### 4.2. Biological Activity

#### 4.2.1. Cell Lines and Culture Conditions

The human skin cancer cell line A 431 and the normal human skin fibroblast cell line BJ were obtained from certified cell repositories. Cells were cultured in DMEM supplemented with 10% fetal bovine serum (FBS), 1% penicillin, and streptomycin, and maintained at 37 °C in a humidified 5% CO_2_ incubator. All experiments were carried out using cells in the exponential growth phase.

#### 4.2.2. MTT Assay for Cell Proliferation [43]

The MTT (3-(4,5-dimethylthiazol-2-yl)-2,5-diphenyl tetrazolium bromide) test was used to evaluate the anticancer activity of N^4^-(5-amino-7-substituted-triazolotriazinepiperazin-1-yl) norfloxacin analogues (**4**–**15**) on the A 431 cell line to confirm cytotoxic activity according to the procedure. The MTT experiment cells were plated at a density of 1 × 10^5^ cells/100 µL in 96-well plates. During a 24 h incubation period, the cells were exposed to N^4^-(5-amino-7-substituted-triazolotriazinepiperazin-1-yl) norfloxacin analogues at concentrations of 31.25, 62.5, 125, 250, 500, and 1000 µg/mL. Wells that contained only serum-free material were chosen as controls. After the 24 h incubation at 37 °C, 20 µL of MTT reagent was added to the wells, which were then incubated for 4 h in the dark. The formazan crystals were dissolved in the wells by adding 200 µL of DMSO. The absorbance was measured at 560 nm. Throughout the experiment’s three repetitions, each concentration was assessed in triplicate. The percentage represents the cell viability of both treated and untreated epidermoid carcinoma cell lines.

% Cell viability = (Absorbance _sample_/Absorbance _control_) × 100

The concentration of N-4-(5-Amino-7-substituted-triazolotriazinepiperazin-1-yl) norfloxacin analogues that resulted in a 50% reduction in cell viability (the half-maximal inhibitory concentration, IC_50_) was calculated. Cell toxicity was determined using the following formula:

%Cell toxicity = ((Absorbance _control_ − Absorbance _sample_)/(Absorbance _control_)) × 100

#### 4.2.3. Cytotoxicity Assay on Normal Cells (BJ)

Based on the initial screening, compounds **14** and **15** exhibited stronger anticancer activity against A 431 skin cancer cells than other synthesized analogues. Therefore, to assess their selectivity and safety profile, these compounds were further evaluated on the normal human skin fibroblast BJ cell line using the MTT assay. This step ensures that the potent anticancer compounds selectively target cancer cells without significant toxicity to normal cells.

#### 4.2.4. Topoisomerase I and II (Topo I/II) Inhibition Assays

The inhibitory activity of the potent compound **14** with high selectivity against cancer cells against Topo I and II enzymes was evaluated using commercial enzyme inhibition kits following the manufacturer’s protocols. For Topo I, the relaxed activity of supercoiled plasmid DNA was measured in the presence of serial concentrations of compound **14** and compared with camptothecin as a positive control. For Topo II, decatenation assays were performed using kinetoplast DNA, with doxorubicin serving as the reference inhibitor. IC_50_ values were calculated from the concentration response curves.

#### 4.2.5. Cell Cycle Analysis (Flow Cytometry)

To determine the effect of compound **14** on cell cycle progression, A 431 cells were seeded in 6-well plates and treated with the IC_50_ concentration of compound **14** for 24 h. Cells were harvested, washed with PBS, and fixed in 70% cold ethanol at 4 °C overnight. Fixed cells were incubated with RNase A (100 µg/mL) and stained with propidium iodide (PI, 50 µg/mL) for 30 min in the dark. DNA content and cell cycle distribution (G0/G1, S, G2/M) were analyzed using a flow cytometer, and results were processed with appropriate analytical software.

#### 4.2.6. Apoptosis Assay (Annexin V-FITC/PI Staining)

Apoptotic cell death induced by compound **14** was evaluated using an Annexin V-FITC/PI apoptosis detection kit. A 431 cells were treated with the compound at its IC_50_ for 24 h, then collected, washed with PBS, and stained with Annexin V-FITC and PI following standard procedures. Cells were analyzed using flow cytometry to determine the percentages of early apoptosis, late apoptosis, necrosis, and viable cells.

#### 4.2.7. Docking Studies

The novel Norfloxacin hybrid **14**, exhibiting the most potent antiproliferative activity, was docked into the active sites of Topo I (PDB ID: 1K4T) and Topo II (7YQ8) to propose a potential mechanism of action and ascertain possible binding modes between the compound and the active sites of both Topo I and Topo II. The molecular structure of compound **14** was generated and optimized using the chemical structure drawing software Marvin Sketch and the molecular modelling application Avogadro. The Protein Data Bank contains the structures of human Topo I (PDB ID: 1K4T) and Topo II (PDB ID: 7YQ8). Molecular docking was performed using (Auto Dock Vina 1.2.0), and Free Discovery Studio Visualizer was used to visualize the best docking configurations.

#### 4.2.8. Statistical Analysis

Different statistical descriptors were used depending on the nature of each experiment. Results from biological replicates (e.g., cytotoxicity assays) are expressed as mean ± SD (standard deviation) to reflect variability across independent experiments. In contrast, experiments involving enzyme inhibition assays and flow cytometry analyses are expressed as mean ± SEM (standard error of the mean), which provides a more accurate indication of the precision of the estimated mean. All experiments were performed in triplicate (n = 3), and statistical significance was determined using Student’s *t*-test, with significance thresholds set at *p* < 0.05, *p* < 0.01, and *p* < 0.001.

## 5. Conclusions

In this study, novel synthesized compounds (**4**–**15**) were tested against A 431 human skin carcinoma cells to determine their anticancer potential. Based on its greater cytotoxic activity than all other compounds studied with an IC_50_ value of 76.7 µg/mL in comparison to the reference medication 5-fluorouracil with an IC_50_ value of 83.7 µg/mL, compound **14** stood out as the most potent among the molecules evaluated. Compound **14** exhibited lower toxicity to normal BJ skin cells than the reference camptothecin with IC_50_ values of 61.8 and 27.8 µM, respectively, indicating greater selectivity of the novel compound **14**. By moderately inhibiting Topo I/II, inducing G0/G1 cell-cycle arrest, and promoting apoptosis, compound **14** exhibited a multi-pathway mechanism of action, as revealed by mechanistic studies. As a result, it is more effective at killing tumour cells and less likely to cause resistance. The results show that compound **14** has great therapeutic potential and is a promising lead structure. To advance its development as a new anticancer drug, additional molecular investigations are recommended including pharmacokinetics, in vivo validation.

## Figures and Tables

**Table 1:** Optimization of the reaction conditions for the synthesis of compound **10** in a one-pot reaction.

Entry	Acidic Cat. (Equiv)	Temperature (°C)	Solvent	Time (h)	Yield (%)
1	AlCl_3_ (1.0 equiv)	R.T.	EtOH	18	Nil
2	Acetic acid (1.0 equiv)	Reflux	EtOH	7	37
3	Trifluoroacetic acid (1.0 equiv)	Reflux	EtOH	7	40
4	AlCl_3_ (1.0 equiv)	Reflux	EtOH	7	43
5	AlCl_3_ (2.0 equiv)	Reflux	EtOH	3	44
6	HCl (1.0 equiv)	R.T.	MeCN	18	50
7	HCl (1.0 equiv)	R.T.	Dioxane	18	60
8	HCl (1.0 equiv)	R.T.	EtOH	18	64
9	HCl (1.0 equiv)	Reflux	EtOH	7	90
**10**	**HCl (1.0 equiv)**	**Reflux**	**EtOH**	**3**	**91**
11	HCl (2.0 equiv)	Reflux	EtOH	3	86

**Table 2:** Percentage of cell viability, cytotoxicity, and IC_50_ values of the in vitro-synthesized compounds (**4**–**15**) tested at different concentrations using the MTT assay.

ID	µg/mL	O.D			Mean O.D	±SD	Viability %	Toxicity %	IC_50_ (µg)
A-431		0.636	0.64	0.644	0.64	0.004	100	0	
4	1000	0.05	0.07	0.06	0.06	0.01	10.57	89.43	118.23
	500	0.1	0.12	0.14	0.12	0.02	19.12	80.88	
	250	0.18	0.20	0.22	0.20	0.02	32.11	67.89	
	125	0.25	0.25	0.43	0.31	0.10	48.61	51.39	
	62.5	0.41	0.42	0.40	0.41	0.01	65.42	34.58	
	31.25	0.51	0.50	0.49	0.50	0.01	79.09	20.91	
5	1000	0.06	0.07	0.08	0.07	0.01	11.4	88.6	131.58
	500	0.10	0.13	0.16	0.13	0.03	21	79	
	250	0.21	0.22	0.23	0.22	0.01	35.1	64.9	
	125	0.32	0.31	0.33	0.32	0.01	51.2	48.8	
	62.5	0.44	0.42	0.43	0.43	0.01	67.3	32.7	
	31.25	0.51	0.52	0.50	0.51	0.01	80.9	19.1	
6	1000	0.09	0.07	0.08	0.08	0.01	13.36	86.64	154.36
	500	0.16	0.15	0.14	0.15	0.01	23.58	76.42	
	250	0.24	0.23	0.25	0.24	0.01	38.17	61.83	
	125	0.36	0.34	0.35	0.35	0.01	55.25	44.75	
	62.5	0.40	0.45	0.50	0.45	0.05	71.43	28.57	
	31.25	0.54	0.53	0.52	0.53	0.01	83.33	16.67	
7	1000	0.06	0.06	0.06	0.06	0	10.1	89.9	120.47
	500	0.13	0.12	0.11	0.12	0.01	19.8	80.2	
	250	0.20	0.22	0.21	0.21	0.01	33.4	66.6	
	125	0.25	0.30	0.38	0.31	0.06	49.9	50.1	
	62.5	0.51	0.40	0.35	0.42	0.08	66.5	33.5	
	31.25	0.54	0.50	0.58	0.51	0.04	80.3	19.7	
8	1000	0.15	0.10	0.08	0.11	0.03	17.24	82.76	208.51
	500	0.10	0.18	0.26	0.18	0.08	29.41	70.59	
	250	0.35	0.27	0.25	0.29	0.05	45.45	54.55	
	125	0.41	0.4	0.39	0.4	0.01	62.5	37.5	
	62.5	0.48	0.49	0.50	0.49	0.01	76.92	23.08	
	31.25	0.50	0.55	0.60	0.55	0.05	86.96	13.04	
9	1000	0.09	0.08	0.04	0.07	0.02	12.3	87.7	136.47
	500	015	0.14	0.13	0.14	0.01	22.5	77.5	
	250	0.29	0.25	0.15	0.23	0.07	37.4	62.6	
	125	0.42	0.35	0.25	0.34	0.08	53.6	46.4	
	62.5	0.52	0.45	0.35	0.44	0.08	69.2	30.8	
	31.25	0.51	0.52	0.53	0.52	0.01	82.1	17.9	
10	1000	0.11	0.07	0.06	0.08	0.02	13.95	86.05	162.03
	500	0.12	0.13	0.20	0.15	0.04	24.45	75.55	
	250	0.30	0.15	0.30	0.25	0.08	39.37	60.63	
	125	0.42	0.38	0.28	0.36	0.07	56.5	43.5	
	62.5	0.38	0.50	0.50	0.46	0.06	71.94	28.06	
	31.25	0.56	0.53	0.50	0.53	0.03	83.99	16.01	
11	1000	0.025	0.022	0.023	0.02	0.001	3.64	96.35	112.9
	500	0.035	0.034	0.027	0.03	0.004	5.00	95.00	
	250	0.083	0.092	0.088	0.08	0.004	13.69	86.30	
	125	0.287	0.26	0.266	0.27	0.01	42.34	57.65	
	62.5	0.531	0.519	0.522	0.52	0.006	81.87	18.12	
	31.25	0.621	0.622	0.617	0.62	0.002	96.87	3.12	
12	1000	0.11	0.10	0.09	0.10	0.01	16.56	83.44	198.45
	500	0.15	0.18	0.21	0.18	0.03	28.41	71.59	
	250	0.32	0.30	0.22	0.28	0.05	44.25	55.75	
	125	0.38	0.39	0.40	0.39	0.01	61.35	38.65	
	62.5	0.44	0.48	0.52	0.48	0.04	76.34	23.66	
	31.25	0.59	0.58	0.48	0.55	0.06	86.21	13.79	
13	1000	0.04	0.08	0.09	0.07	0.02	11.8	88.2	134.67
	500	0.11	0.13	0.15	0.13	0.02	21.8	78.2	
	250	0.23	0.26	0.20	0.23	0.03	36.2	63.8	
	125	0.36	0.30	0.33	0.33	0.03	52.3	47.7	
	62.5	0.40	0.43	0.46	0.43	0.03	68	32	
	31.25	0.48	0.51	0.54	0.51	0.03	81.2	18.8	
14	1000	0.022	0.03	0.026	0.02	0.004	4.06	95.93	76.7
	500	0.028	0.025	0.029	0.02	0.002	4.27	95.72	
	250	0.07	0.062	0.066	0.06	0.004	10.31	89.68	
	125	0.171	0.157	0.185	0.17	0.014	26.71	73.28	
	62.5	0.378	0.354	0.36	0.36	0.012	56.87	43.12	
	31.25	0.554	0.531	0.538	0.54	0.011	84.53	15.46	
15	1000	0.034	0.033	0.028	0.03	0.003	4.94	95.05	83.6
	500	0.032	0.028	0.043	0.03	0.007	5.36	94.63	
	250	0.055	0.037	0.044	0.04	0.009	7.08	92.91	
	125	0.165	0.19	0.177	0.17	0.012	27.70	72.29	
	62.5	0.387	0.389	0.402	0.39	0.008	61.35	38.64	
	31.25	0.626	0.638	0.633	0.63	0.006	98.80	1.19	
5-FU (Standard)	1000	0.018	0.018	0.022	0.01	0.002	3.78	96.21	83.7
	500	0.02	0.017	0.018	0.01	0.001	3.58	96.41	
	250	0.108	0.1	0.013	0.07	0.052	14.41	85.58	
	125	0.088	0.093	0.09	0.09	0.002	17.67	82.32	
	62.5	0.325	0.333	0.362	0.34	0.019	66.53	33.46	
	31.25	0.465	0.472	0.459	0.46	0.006	91.06	8.93	

**Table 3:** In vitro cytotoxicity of compounds **14** and **15** expressed as half-maximal inhibitory concentration (IC_50_, µM ± SD) in comparison with the standard cytotoxic agent camptothecin (number of experiments n = 3).

Compound	In Vitro Cytotoxicity (IC_50_)	*p* Value
	BJ Normal Cell Line (µM ± SD)	
**14**	61.8 ± 1.9	<0.001
**15**	43.8 ± 1.4	<0.001
**camptothecin**	27.8 ± 0.87	<0.001

**Table 4:** Topoisomerase I and II inhibitory activities of compound **14** expressed as IC_50_ in comparison with the reference inhibitors camptothecin (Topo I) and doxorubicin (Topo II) (number of experiments n = 3).

Compound	Topoisomerase I IC_50_ µM ± SD	Topoisomerase II IC_50_ µM ± SD	*p* Value
**14**	17.5 ± 0.59	17.3 ± 1.02	<0.001
**doxorubicin**	NT	1.14 ± 0.07	<0.001
**camptothecin**	2.485 ± 0.08	NT	<0.001

NT: not tested.

**Table 5:** DNA content analysis of A 431 skin cancer cells treated with the IC_50_ concentration of compound **14** compared with DMSO-treated cells (negative control). The table shows the percentage distribution of cells across the G0/G1, S, and G2/M phases of the cell cycle.

Compound	DNA Content		
	% G0/G1	% S	% G2/M
**14**/A 431	64.09	27.54	8.37
**DMSO**/A 431	58.18	29.61	12.21

**Table 6:** Apoptosis and necrosis levels in A 431 skin cancer cells treated with the IC50 concentrations of compound 14, compared with untreated cells (A 431–DMSO, negative control). The table presents the percentages of early apoptosis, late apoptosis, and necrosis as determined by Annexin V-FITC/PI staining.

Compound	Apoptosis			Necrosis
	Total	Early	Late	
**14/**HCT116	**22.26**	7.22	15.04	4.77
**DMSO/**HCT116	**0.79**	0.61	0.18	1.85

**Table 7:** Molecular docking data for compound **14** and camptothecin against human Topoisomerase I (PDB ID: 1K4T).

Compound	Target	Binding Affinity (Kcal/mol)	Amino Acid Residue/DNA Nucleotide Base	Types of Interaction	Bond Length (A°)
**14**	Topo I	−7.9	ARG 375	H-bond	2.54
			TRP 416	H-bond	2.04
			ASN 419	H-bond	2.59
			GLU 418	H-bond	2.38
			ARG 375	Pi-anion	3.54
			TGP 11	Pi-anion	4.23
			DT 10	Pi-anion	3.33
			LYS 374	Pi-Alkyl	4.12
			ILE 420	Pi-Alkyl	5.06
			PHE 361	Pi-Alkyl	5.14
			DC 112	Pi-Alkyl	4.26
			DA 113	Pi-Pi	4.37
**camptothecin**	Topo I	−8.5	DC 112	Pi-Pi	4.56
			DA 113	Pi-Pi	3.89
			TGP 11	Pi-Pi	4.13
			DT 10	H-bond	2.43
			ASN 722	H-bond	2.11
			ARG 364	H-bond	2.07
			ASP 533	H-bond	1.92

**Table 8:** Molecular docking data for compound **14** and etoposide against human Topo IIβ (PDB: ID 7YQ8).

Compound	Target	Binding Affinity (Kcal/mol)	Amino Acid Residue/DNA Nucleotide Base	Types of Interaction	Bond Length (A°)
**14**	Topo IIβ	−9.2	DC 28	Pi-Pi	3.56
			DC 22	Pi-Pi	4.77
			DG 27	Pi-Pi	4.69
			MET 787	Pi-Alkyl	2.03
			ASN 791	H-bond	1.89
			GLN 783	H-bond	2.03
			ARG 508	Pi-cation	3.11
			LYS 819	Pi-cation	3.86
			GLN 794	H-bond	1.91
			GLY 818	H-bond	2.34
***Doxorubicin***	Topo IIβ	−9.3	DC 22, DG 27	Pi-Alkyl	2.73
			MET 787	Pi-Alkyl	3.51
			DC 28	Pi-Pi	3.98
			DG 28	H-bond	2.45
			GLN 783, ARG 508	H-bond	2.09

## Data Availability

The original contributions presented in this study are included in the article/Supplementary Materials. Further inquiries can be directed to the corresponding authors.

## Supplementary Materials

The following supporting information can be downloaded at https://www.mdpi.com/article/10.3390/ph19050657/s1.
