# Supplementary material for: Novel N-4-(5-Amino-7-substituted-triazolotriazinepiperazin-1-yl) Norfloxacin Analogues Exhibit Potent and Selective Anticancer Activity via Topoisomerase Inhibition, Cell-Cycle Arrest, and Apoptosis in A 431 Skin Carcinoma Cells
Source: Pharmaceuticals (Basel). 2026 Apr 22;19(5):657. doi: 10.3390/ph19050657 (PMC13209770; doi:10.3390/ph19050657)
Supplement: Supplementary file 1 [file pharmaceuticals-19-00657-s001.zip › pharmaceuticals-4248753-supplementary.pdf]

Supplementary data

Novel N-4-(5-Amino-7-substituted-triazolotriazinepiperazin-1-yl) norfloxacin Analogues Exhibit Potent and Selective Anticancer Activity *via* Topoisomerase Inhibition, Cell-Cycle Arrest, and Apoptosis in A 431 Skin Carcinoma Cells

Ahmed M. El-Saghier [1, *], Amany M. Hamed [1], Laila Abosella [1,2], Stefan Bräse [3, *], Hossameldin A. Aziz [4, 5]

[1]Chemistry Department, Faculty of Science, Sohag University, 282524 Sohag, Egypt.
[1,2]Medicinal Chemistry Department, Faculty of Pharmacy, Sabratha University, Sabratha, Libya.
[3]Institute for Biological and Chemical System, Karlsruhe Institute of Technology, 76131 Karlsruhe, Germany.
[4]Pharmaceutical Chemistry Department, Faculty of Pharmacy, New Valley University, New Valley, 72511, Egypt.
[5]Pharmaceutical Chemistry Department, Faculty of Pharmacy, New Valley National University, New Valley, 72511, Egypt

**To whom correspondence should be addressed:*

Ahmed M. El-Saghier; E-mail: el.saghier@science.sohag.edu.eg

Stefan Bräse; E-mail address: stefan.braese@kit.edu

Spectral analysis for the newly synthesized compounds 4-15

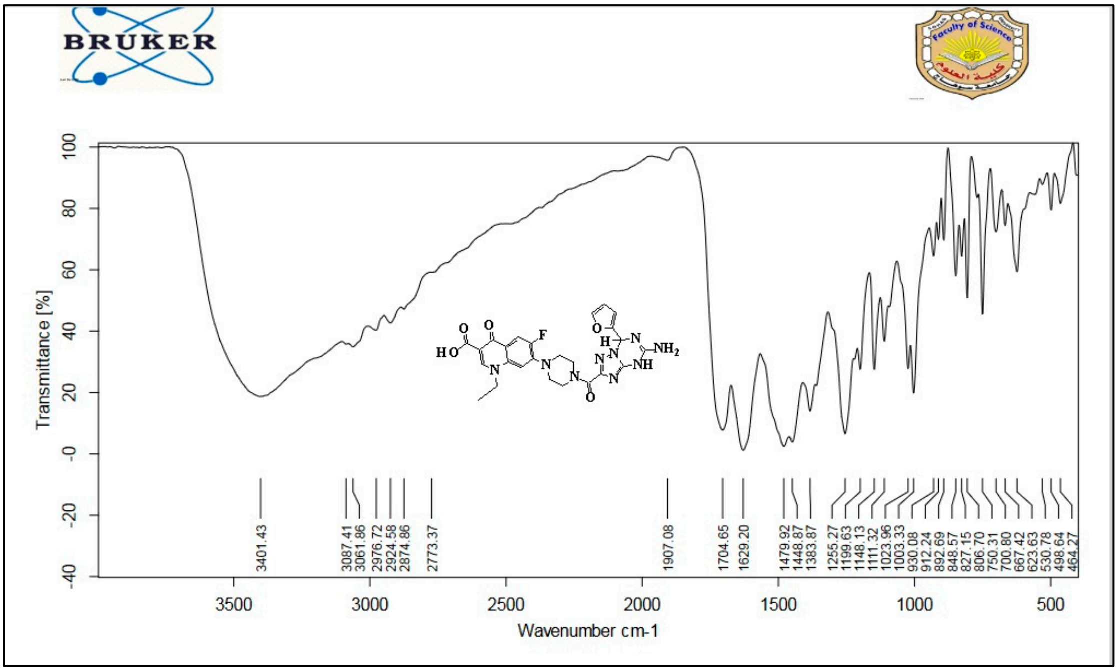

FigureS1: IR Spectrum of compound **4**

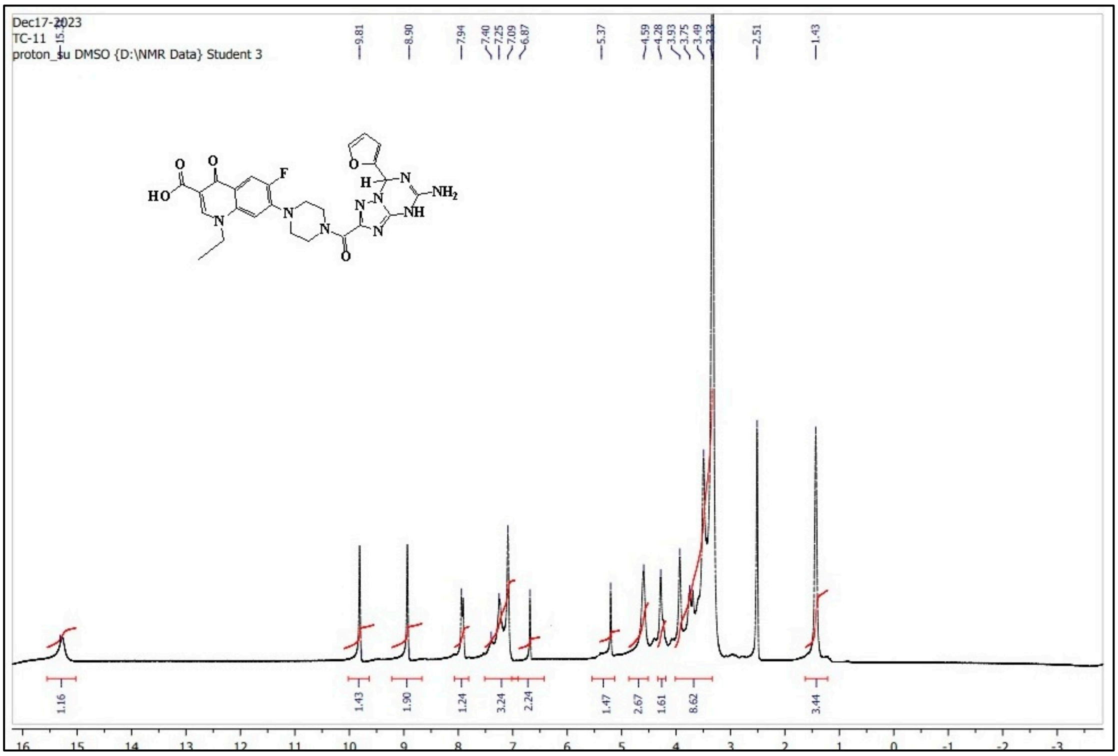

Figure S2: [1] HNMR Spectrum of compound **4**

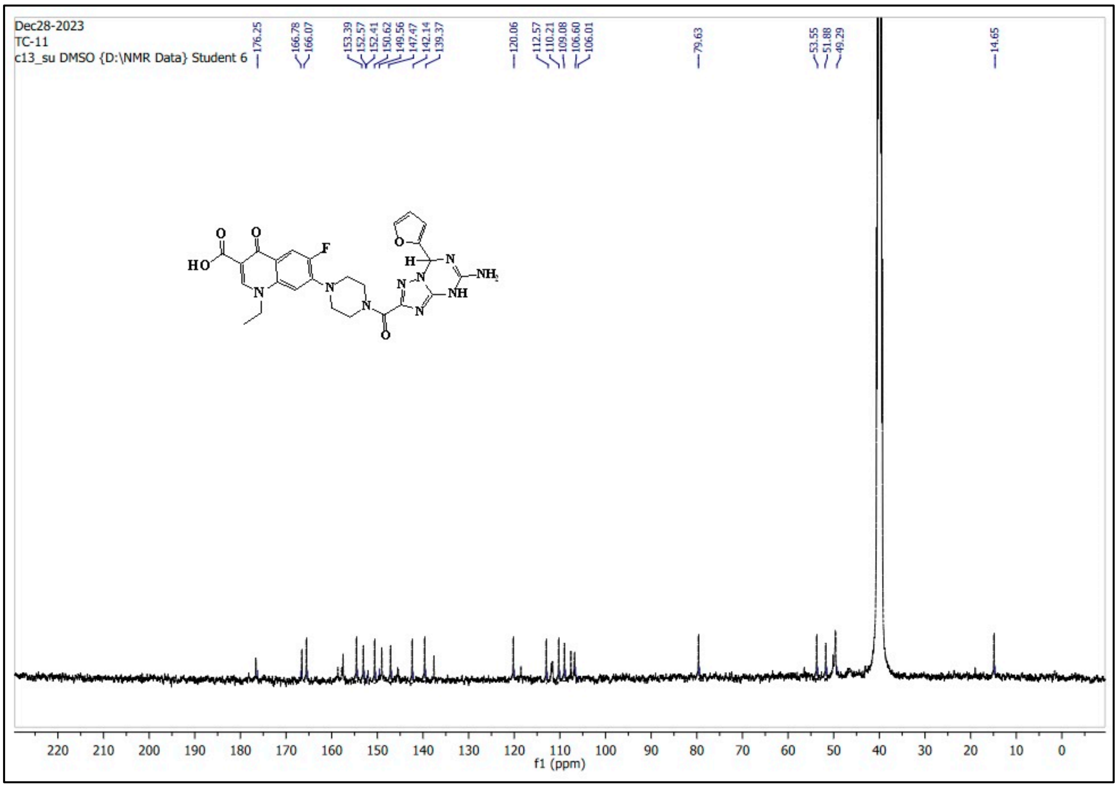

Figure S3: [13] CNMR Spectrum of compound **4**

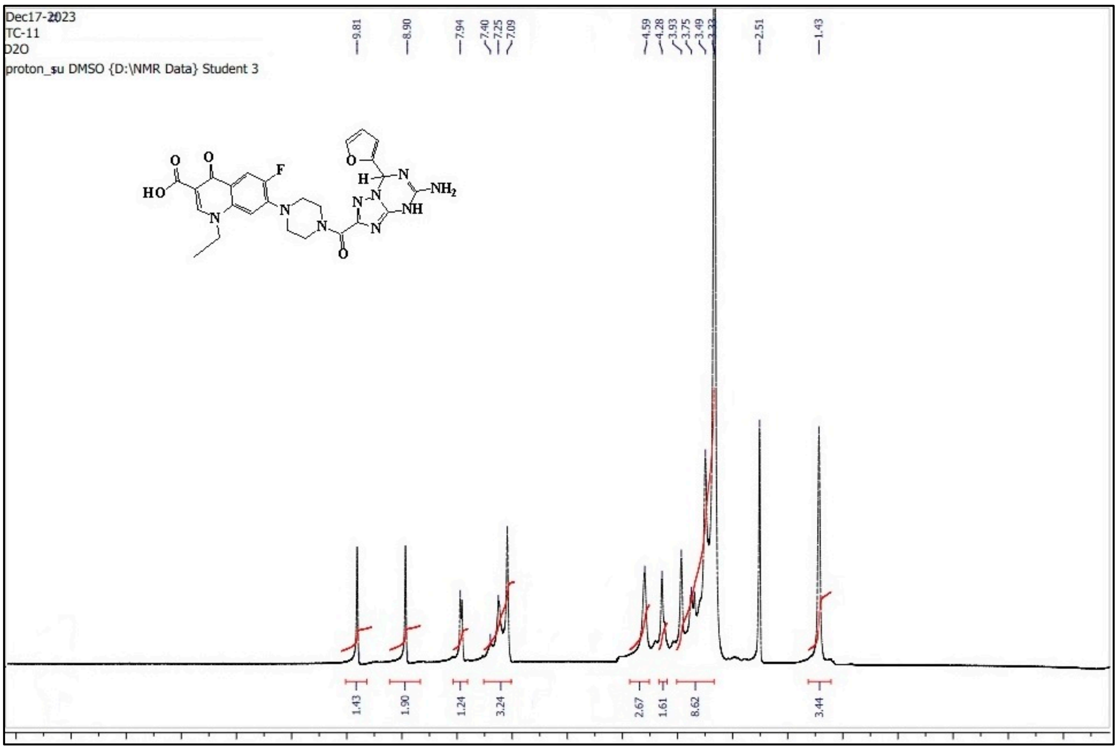

Figure S4: D_2O NMR spectrum for compound **4**

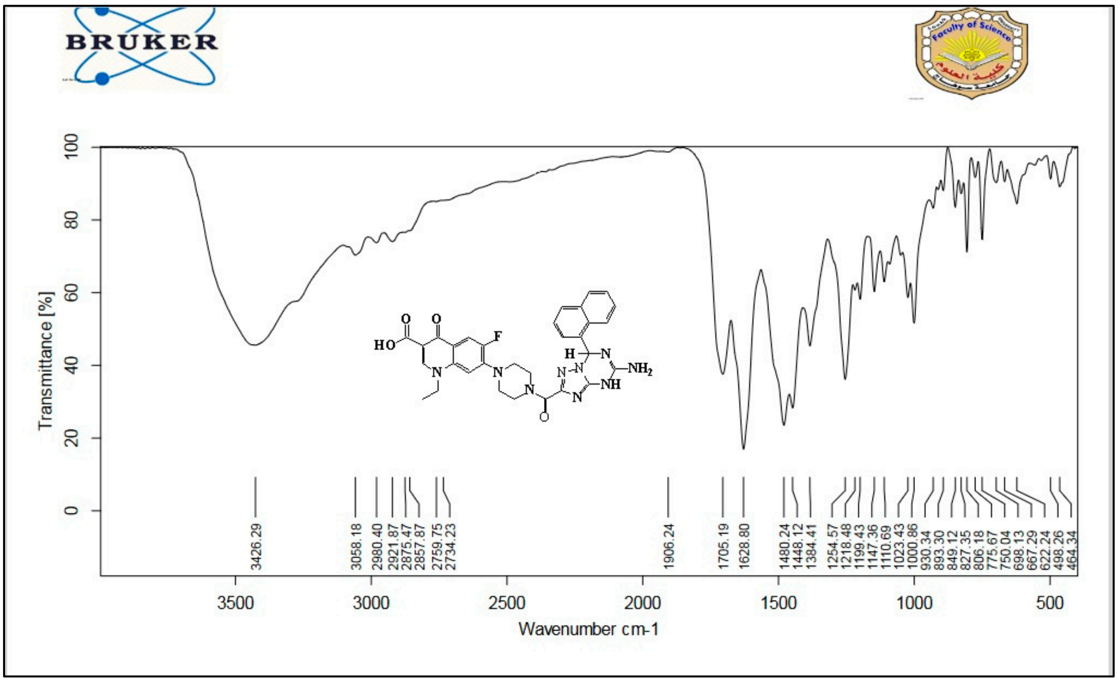

Figure S5: IR Spectrum of compound **5**

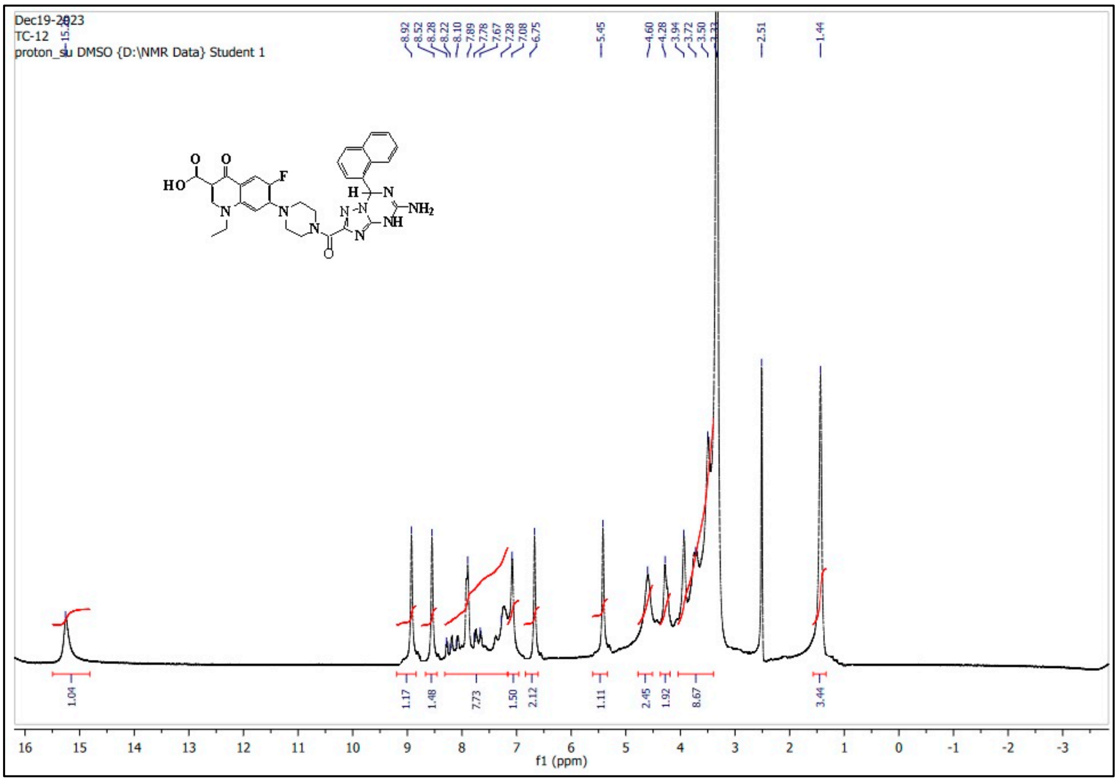

Figure S6: [1] HNMR Spectrum of compound **5**

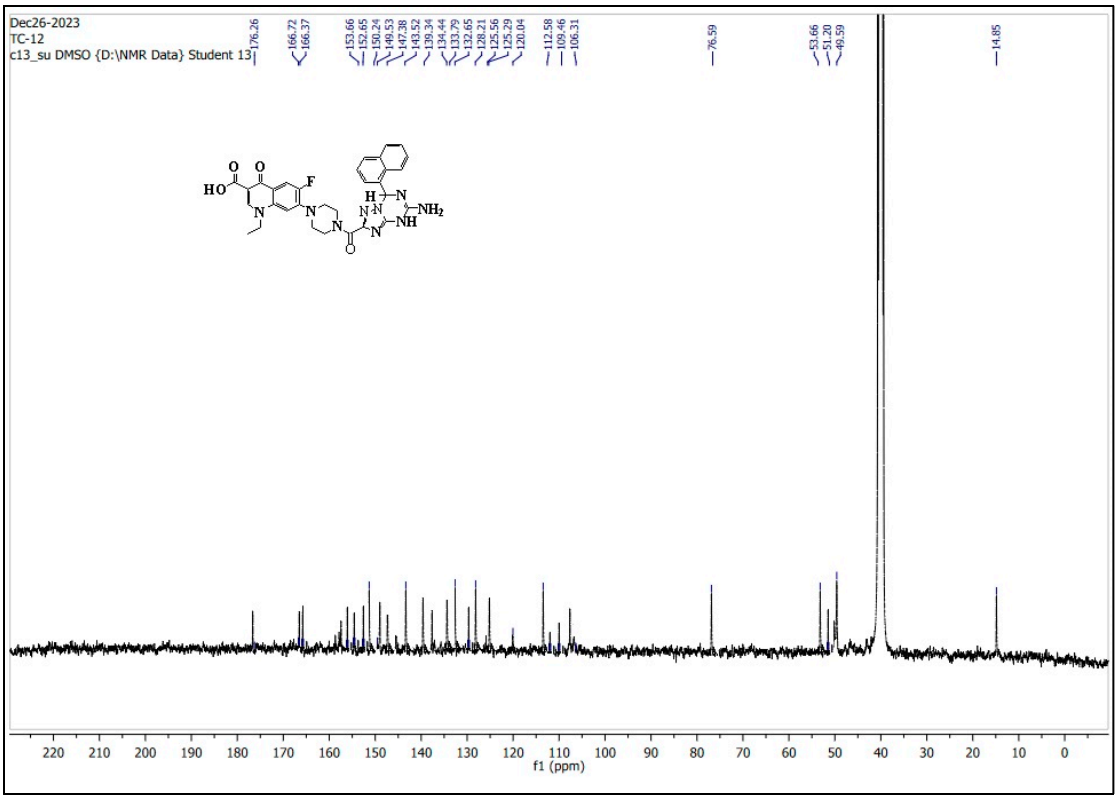

Figure S7: ^{13} CNMR Spectrum of compound **5**

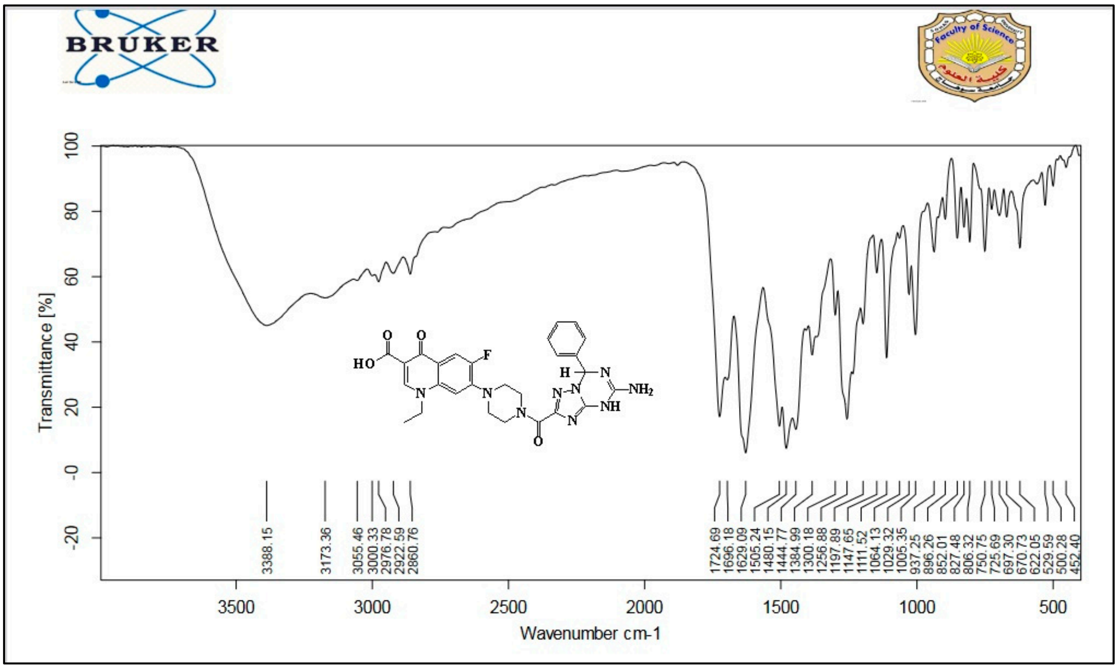

Figure SS8: IR Spectrum of compound **6**

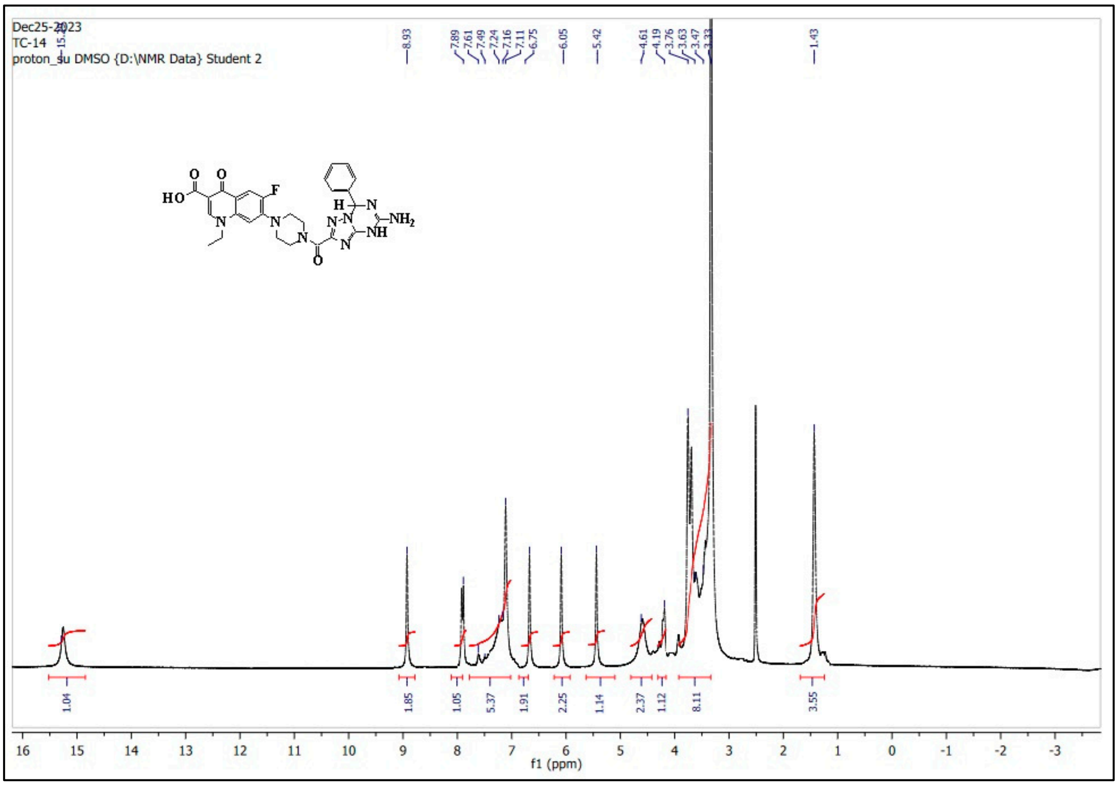

Figure S9: [1] HNMR Spectrum of compound **6**

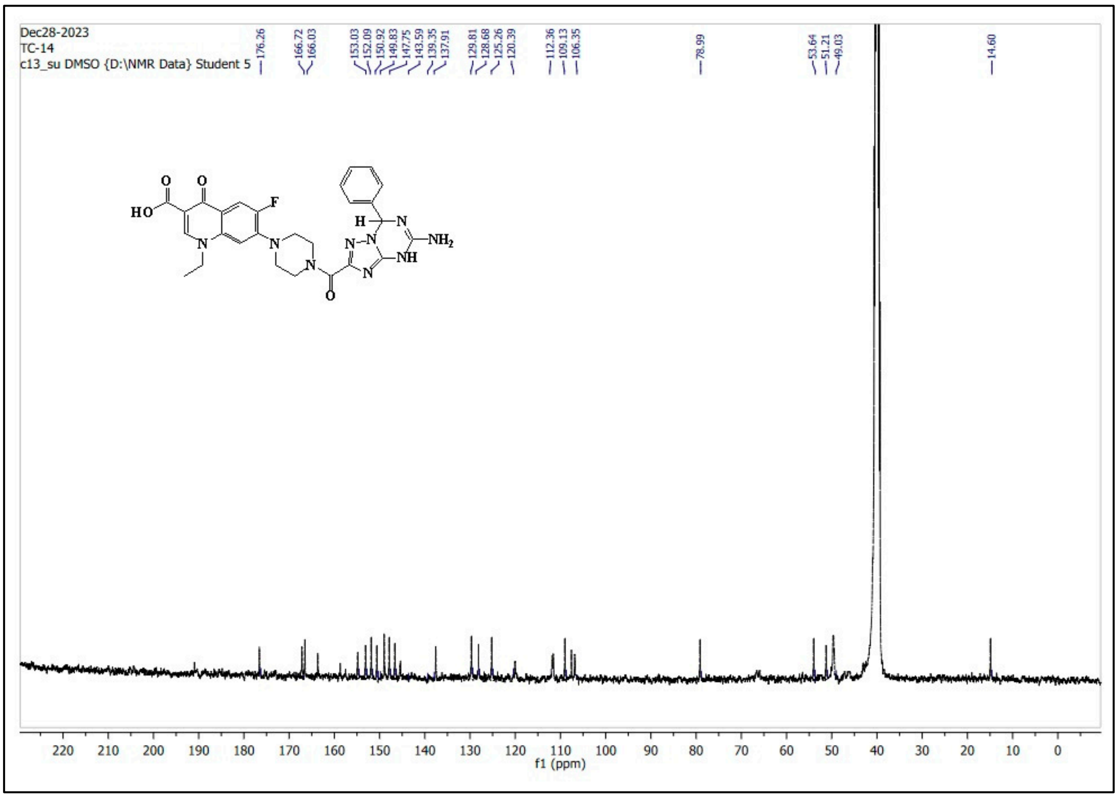

Figure S10: [13] CNMR Spectrum of compound **6**

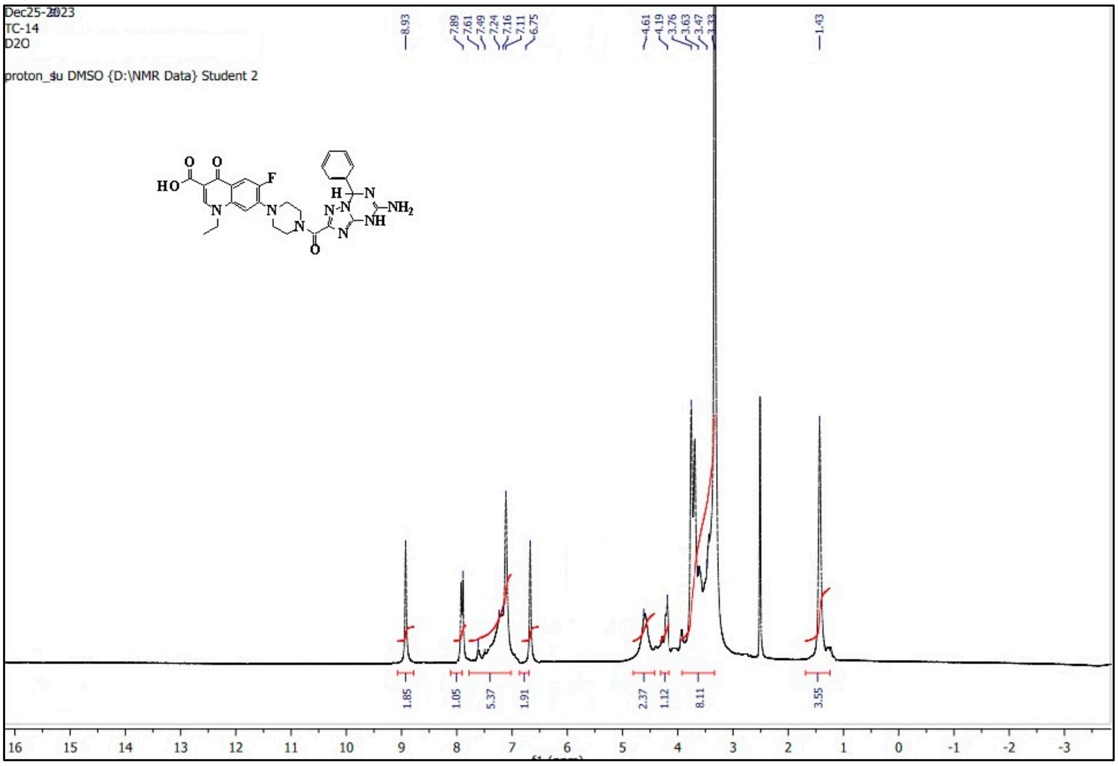

Figure S11: D_2O NMR spectrum for compound **6**

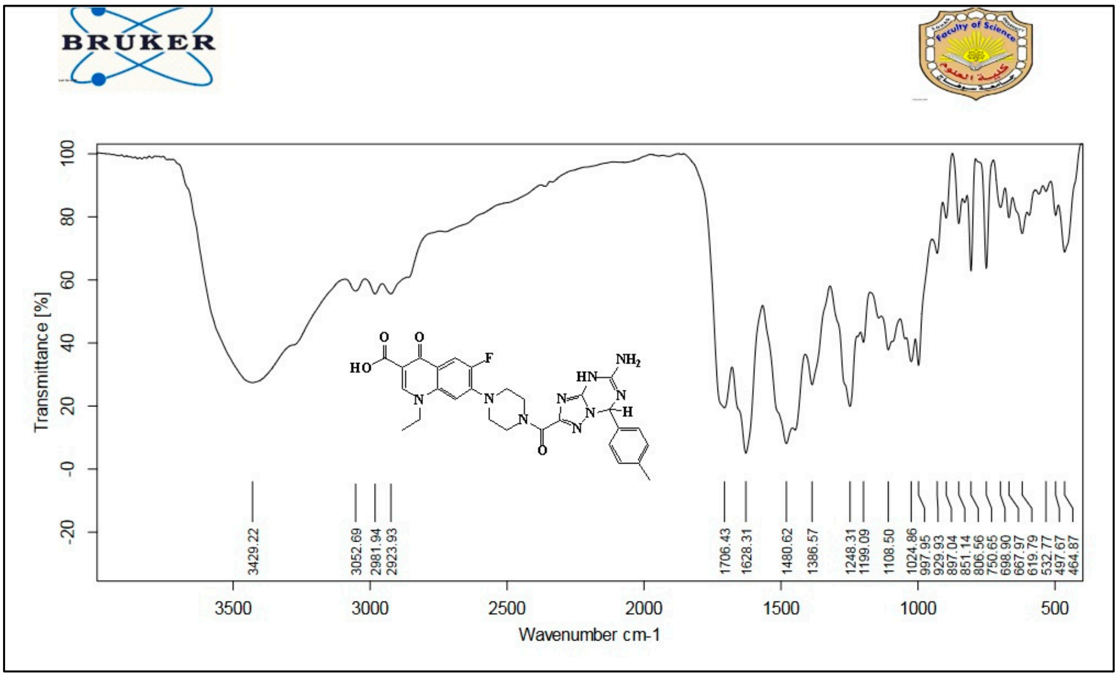

Figure S12: IR Spectrum of compound **7**

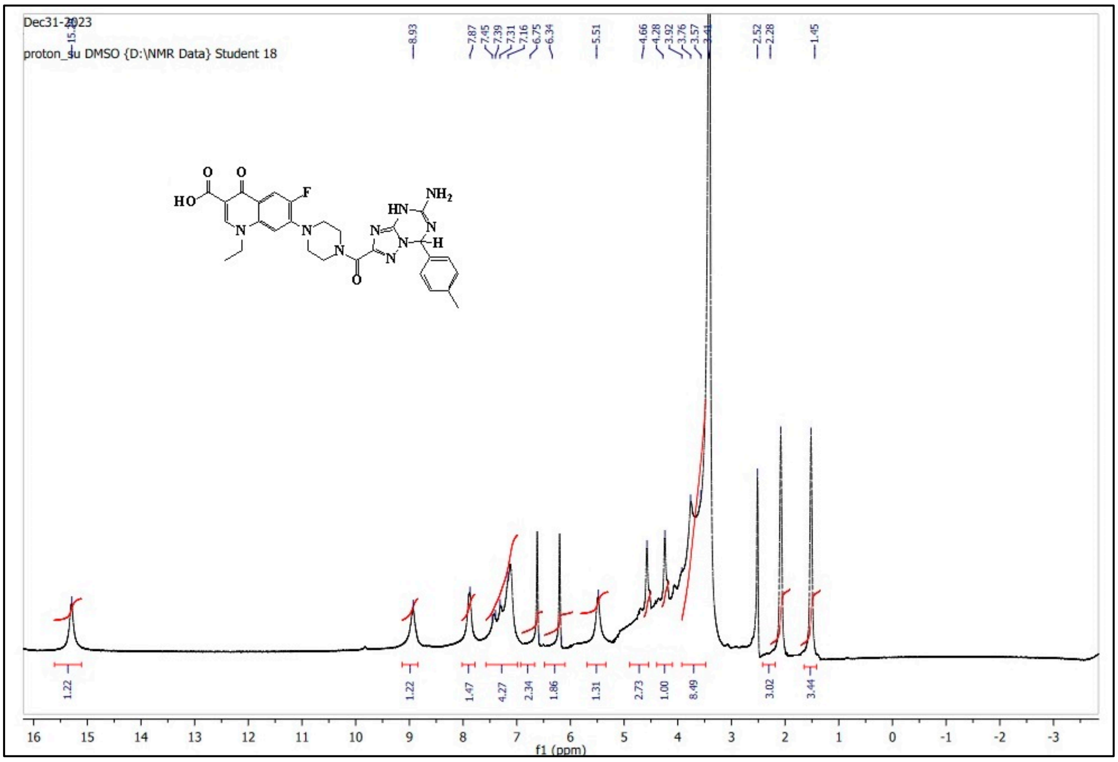

Figure S13: ^{1} HNMR Spectrum of compound **7**

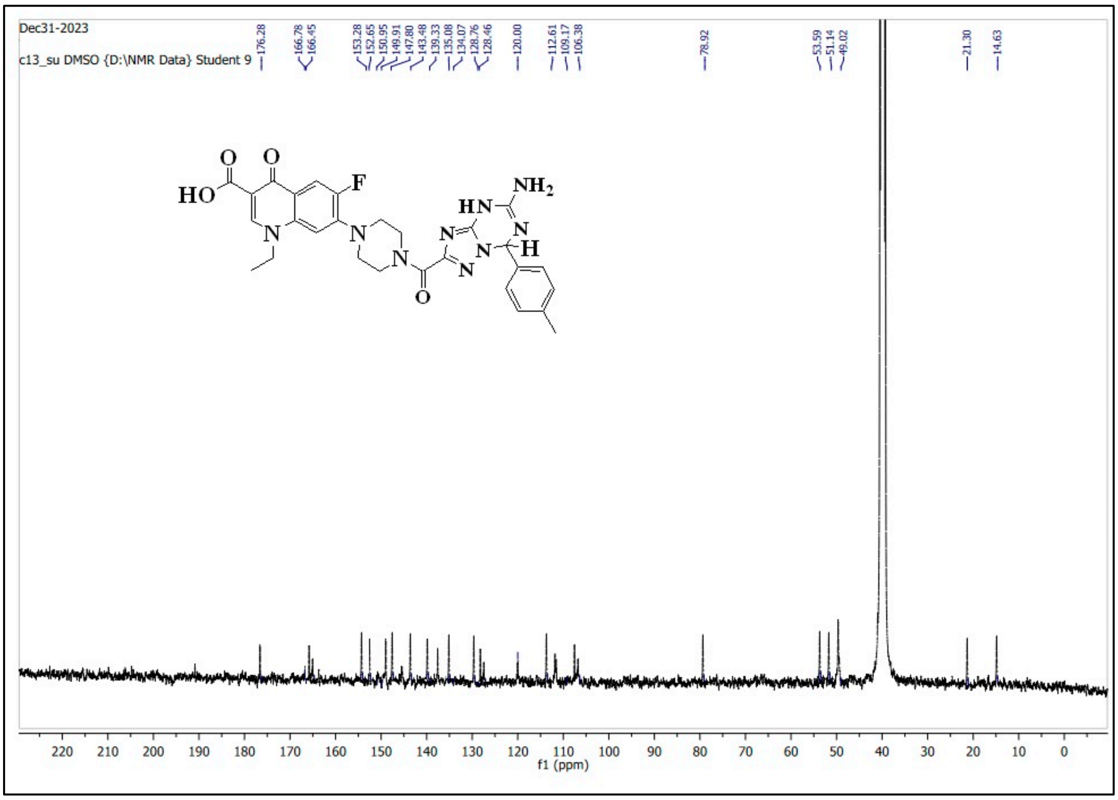

Figure S14: ^{13} CNMR Spectrum of compound **7**

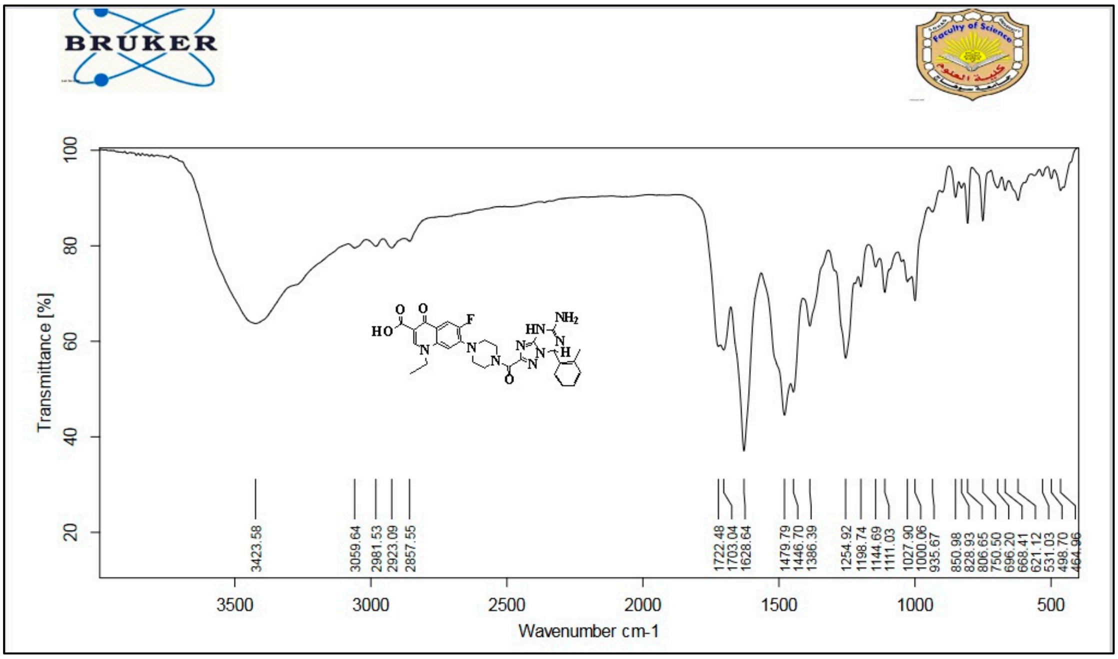

Figure SS15: IR Spectrum of compound **8**

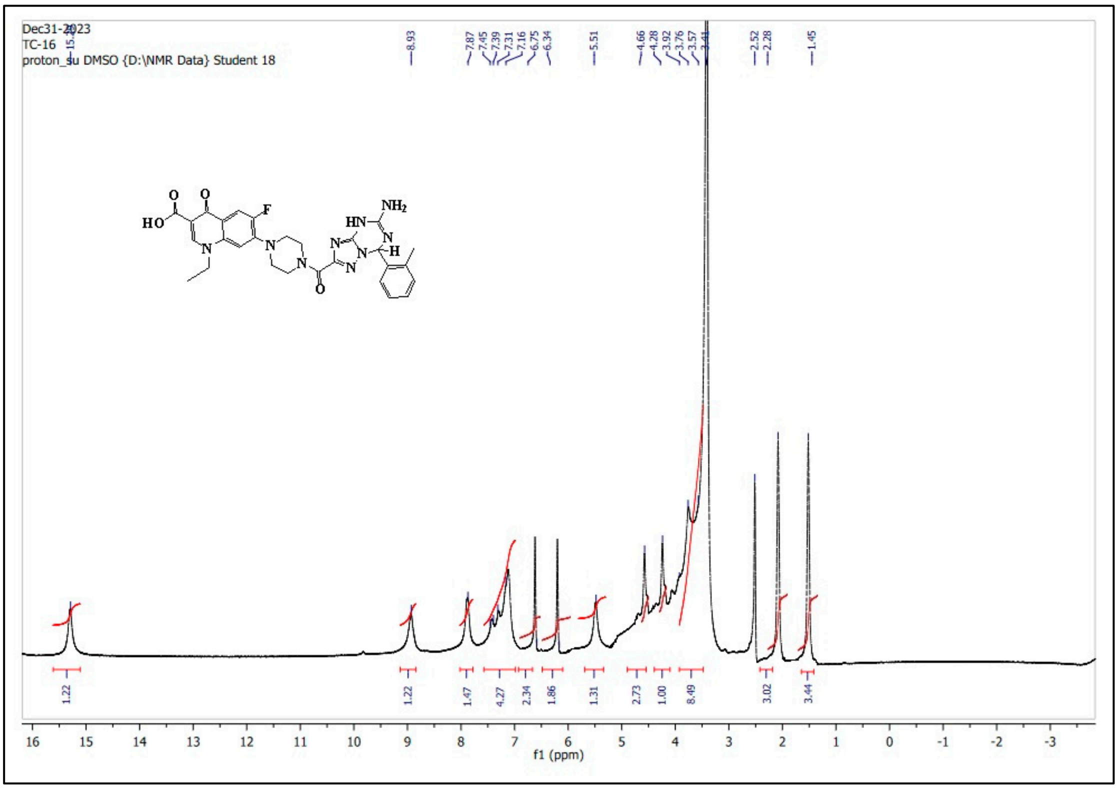

Figure S16: [1] HNMR Spectrum of compound **8**

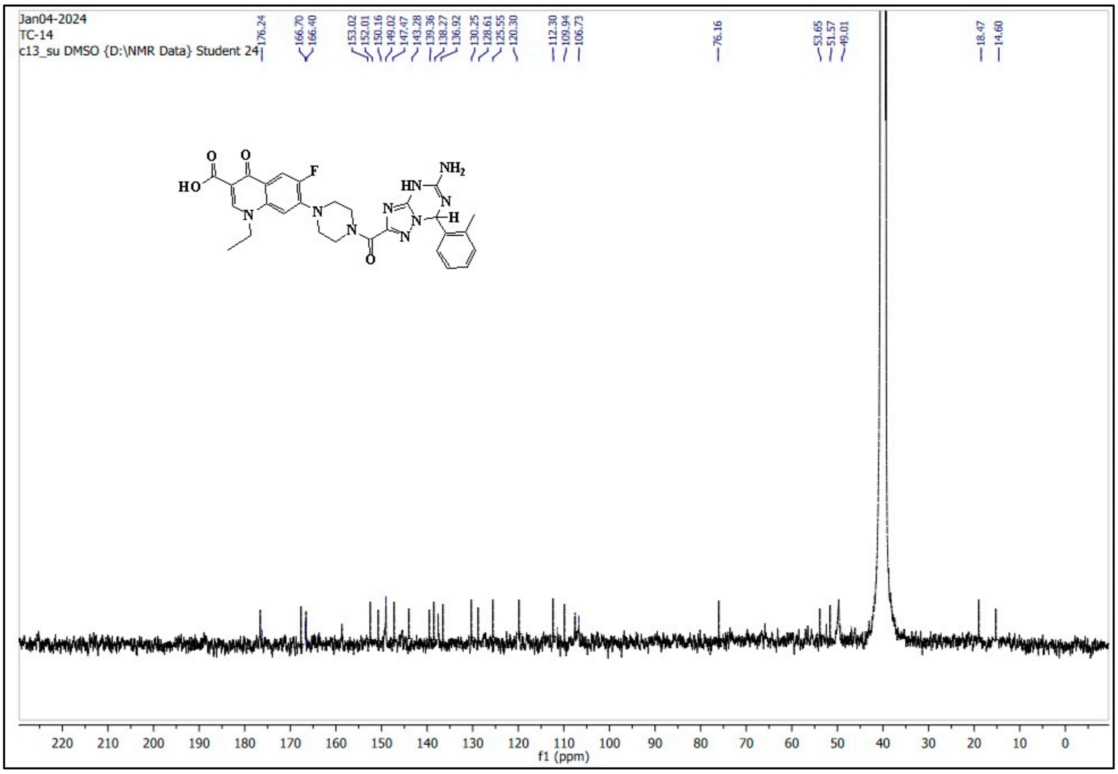

Figure S17: ^{13} CNMR Spectrum of compound **8**

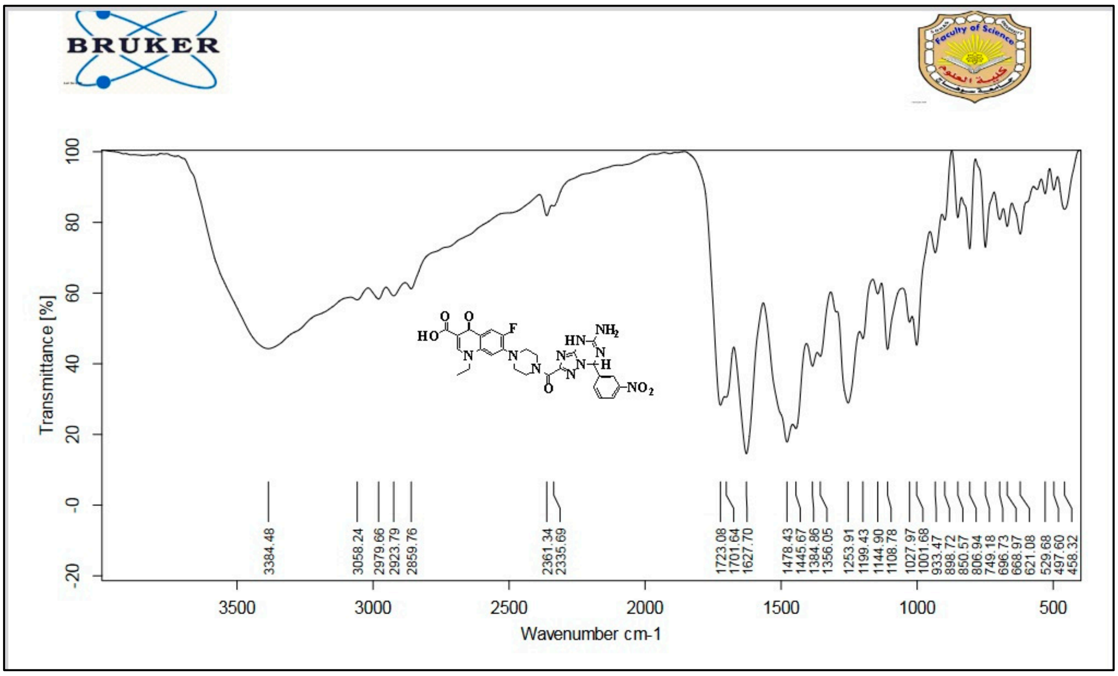

Figure S18: IR Spectrum of compound **9**

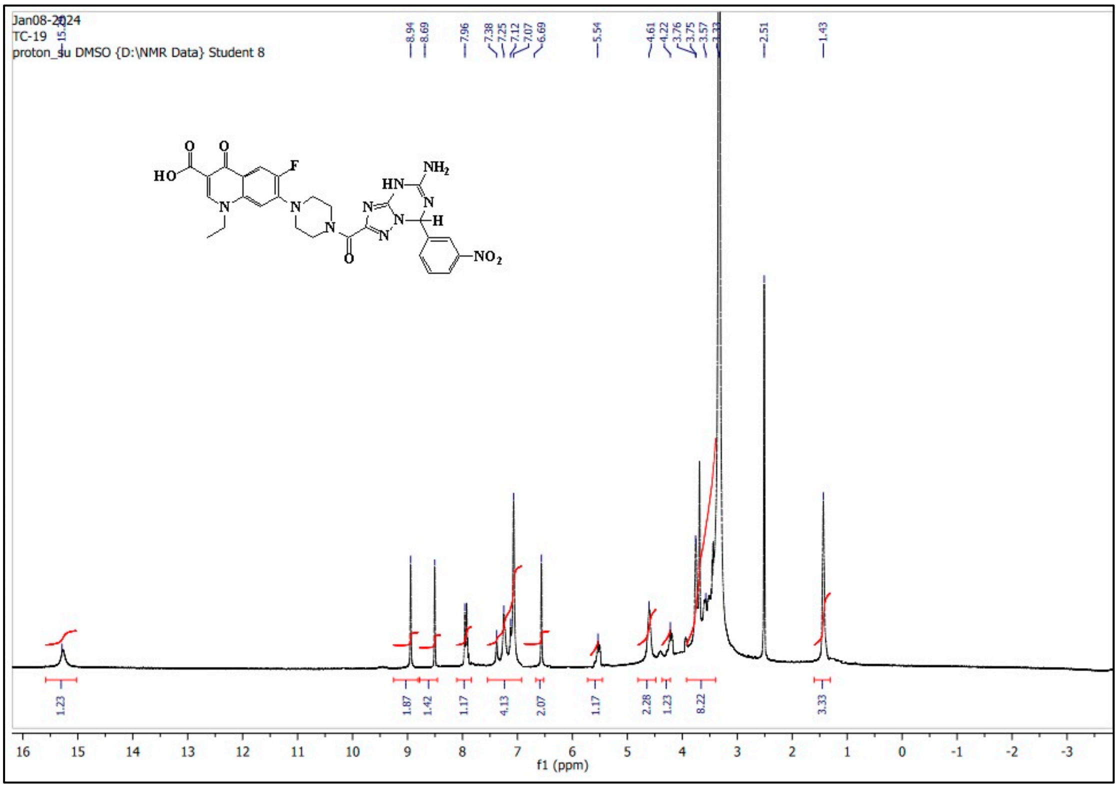

Figure S19: [1] HNMR Spectrum of compound **9**

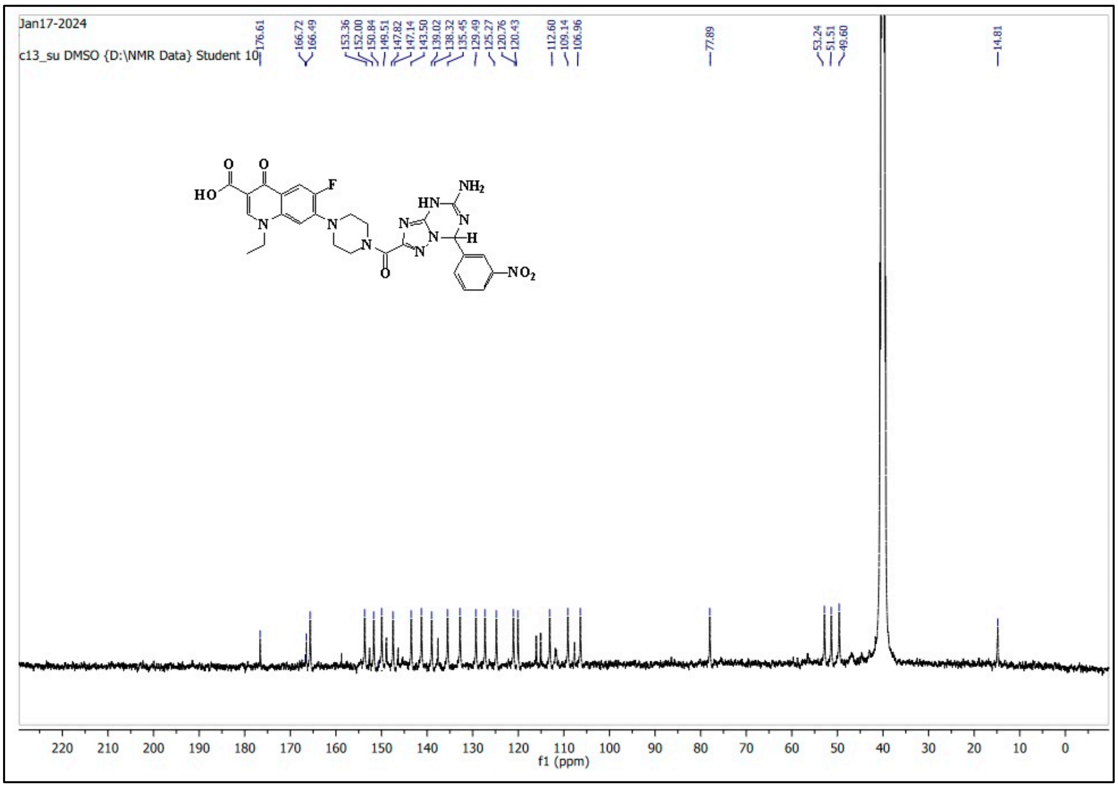

Figure S20: [13] CNMR Spectrum of compound **9**

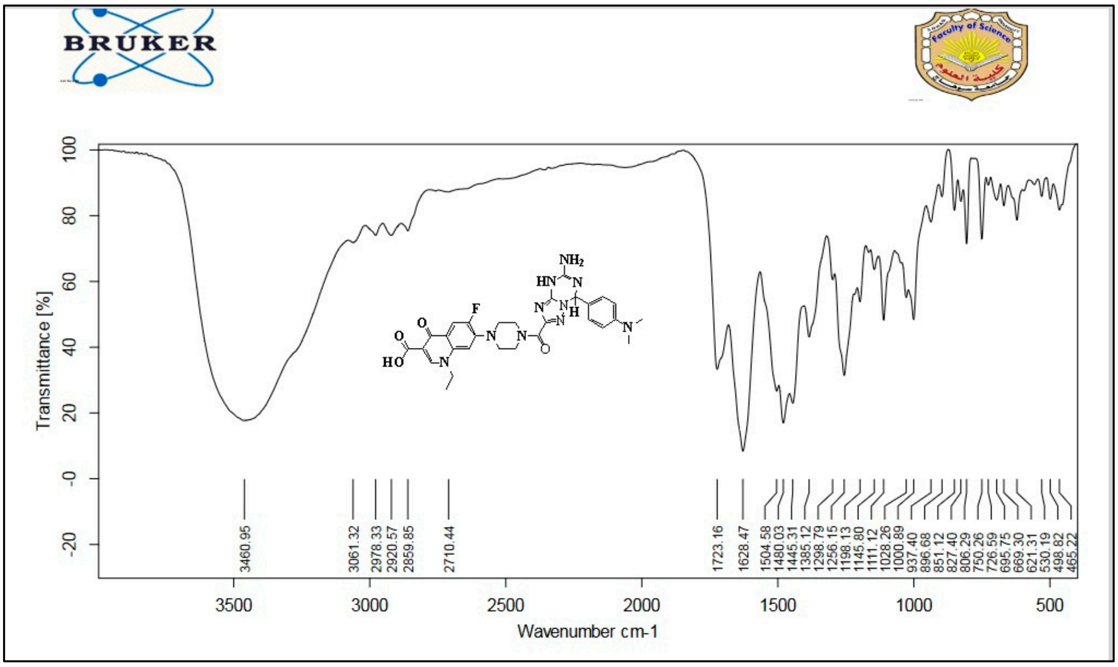

Figure S21: IR Spectrum of compound **10**

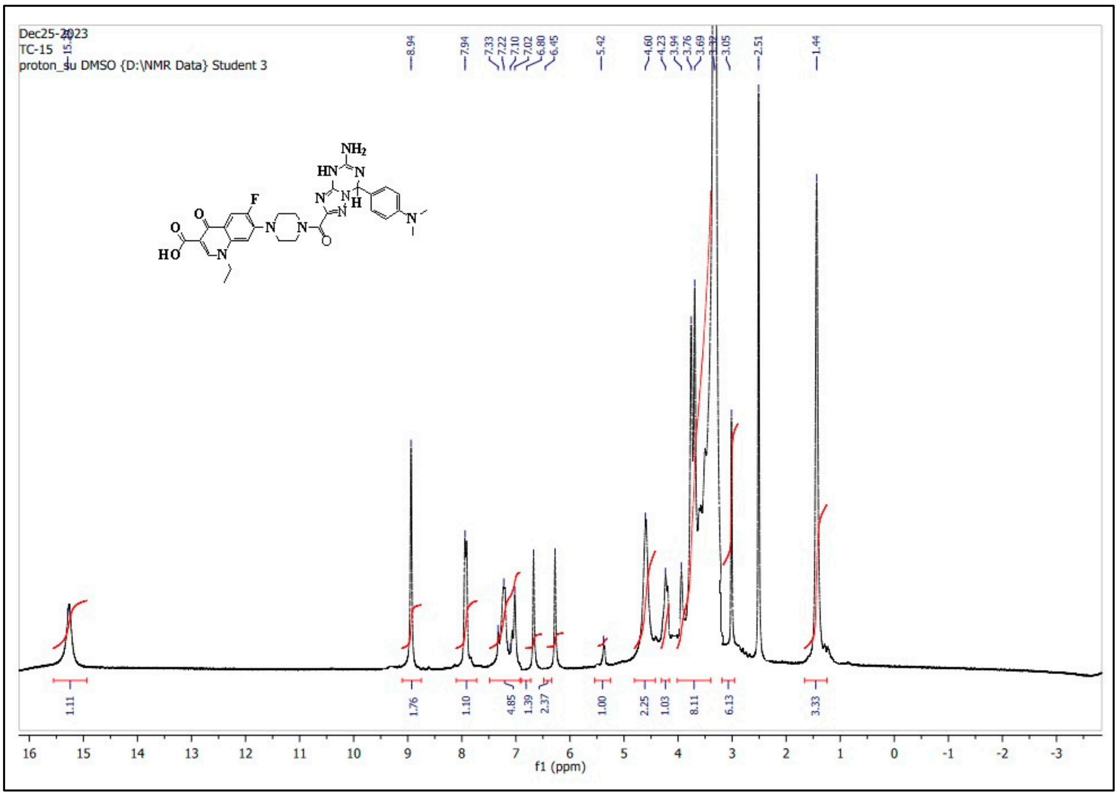

Figure S22: [1] HNMR Spectrum of compound **10**

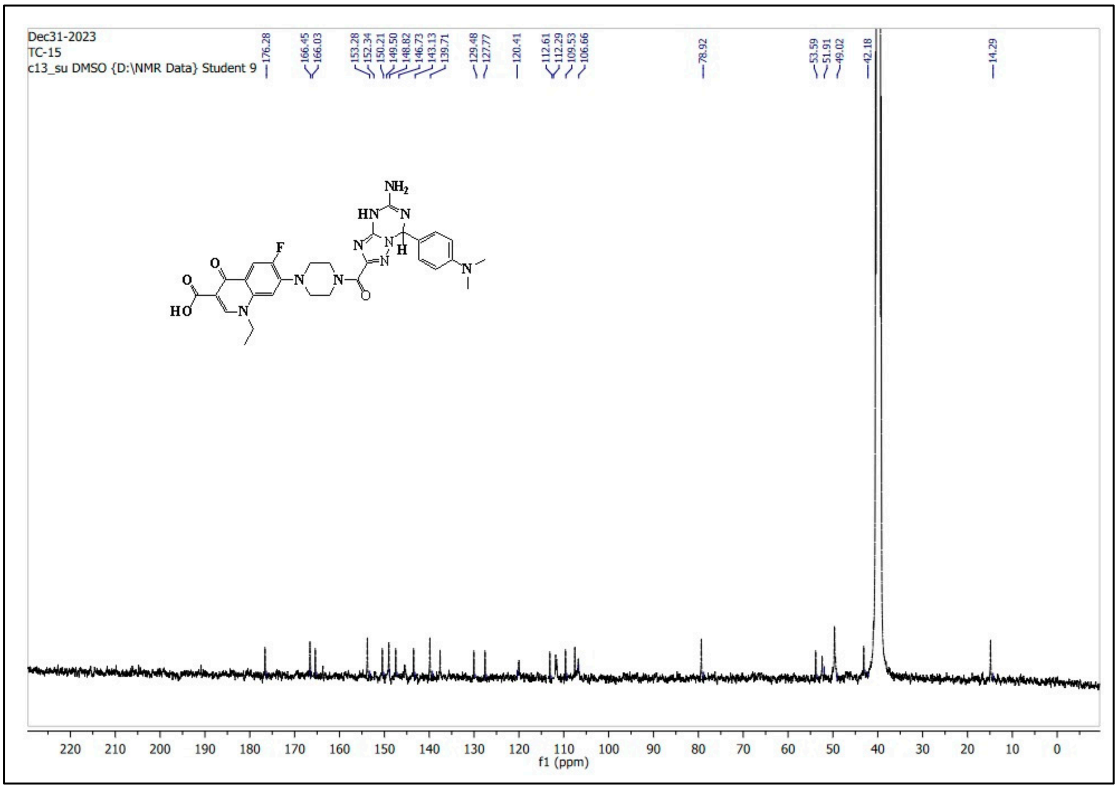

Figure S23: ^{13} CNMR Spectrum of compound **10**

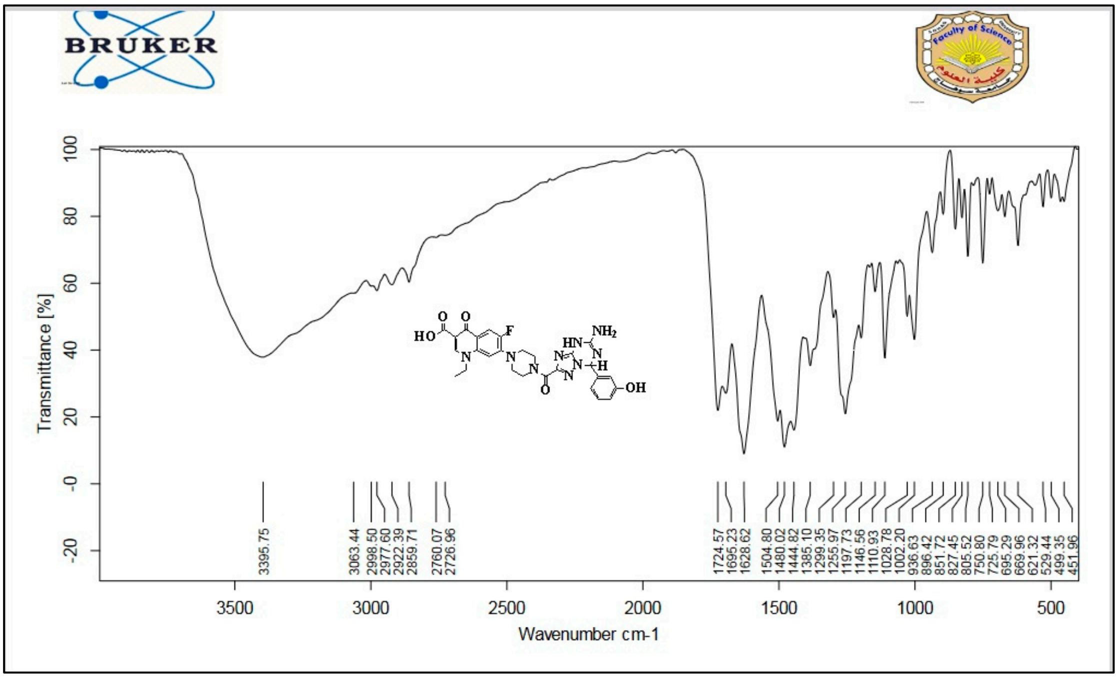

Figure S24: IR Spectrum of compound **11**

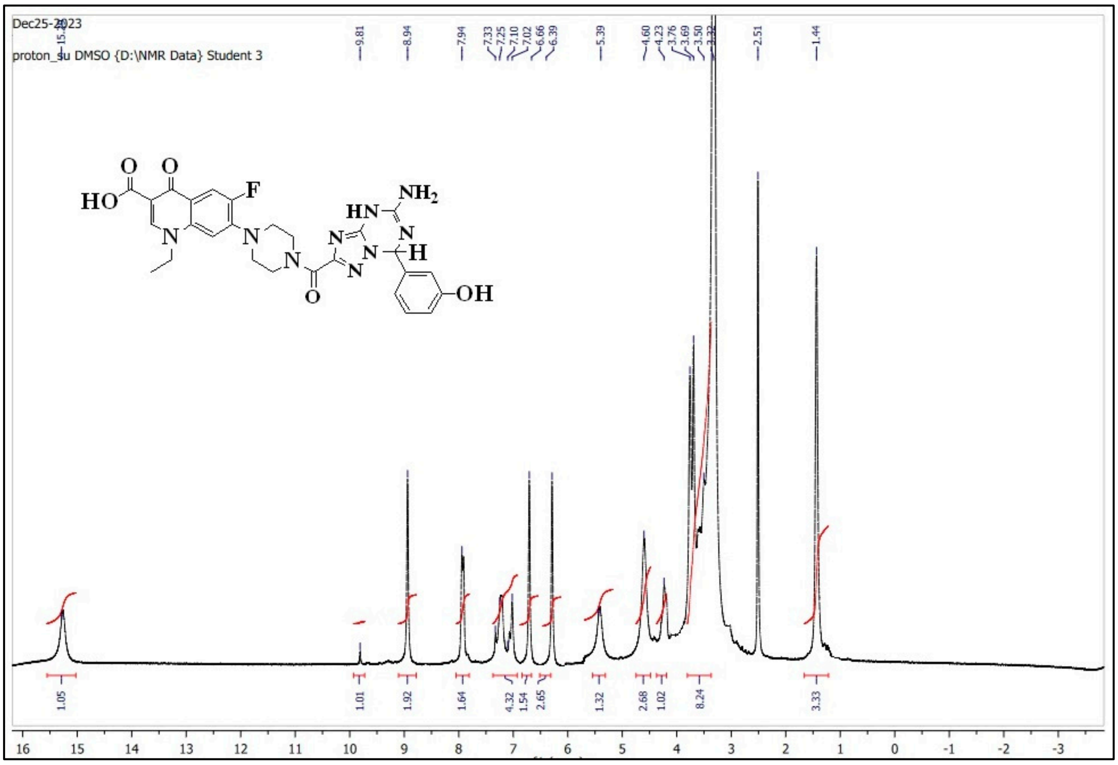

Figure S25: [1] HNMR Spectrum of compound **11**

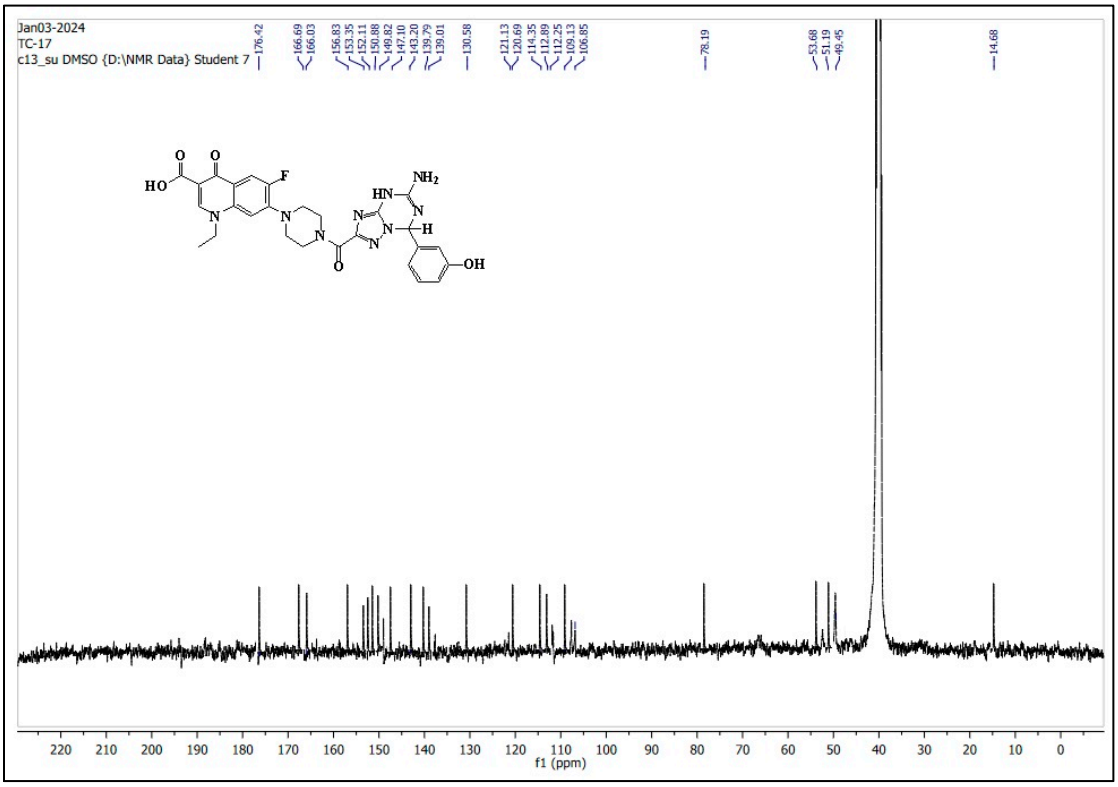

Figure S26: [13] CNMR Spectrum of compound **11**

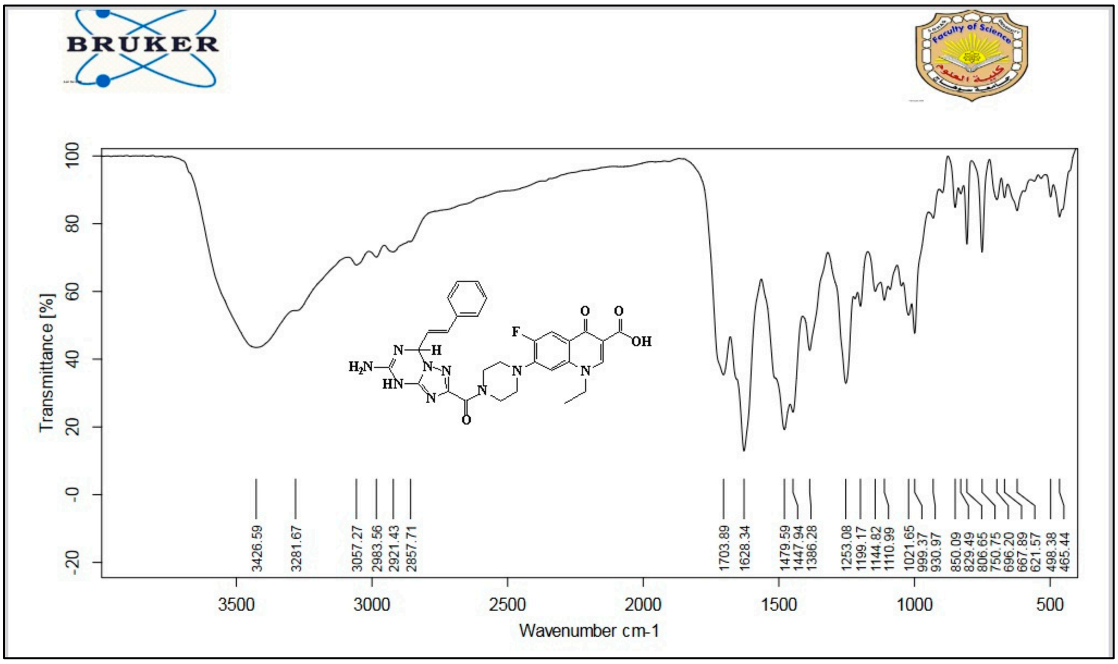

Figure S27: IR Spectrum of compound **12**

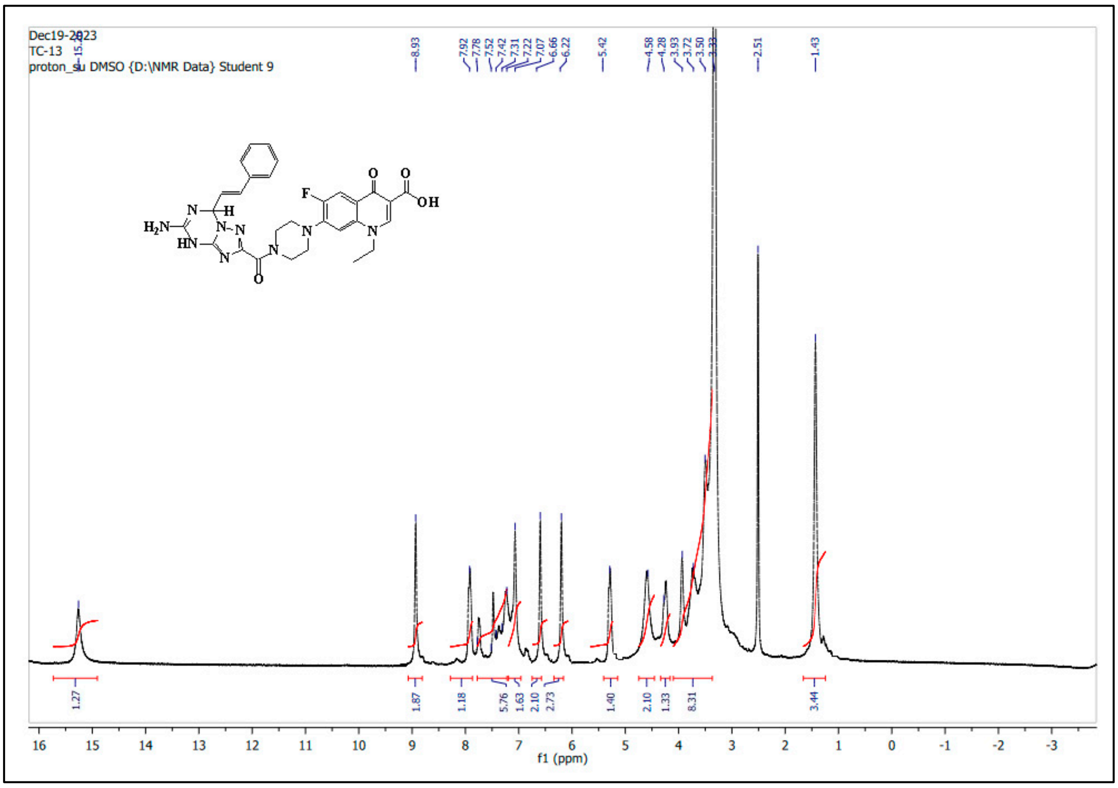

Figure S28: [1] HNMR Spectrum of compound **12**

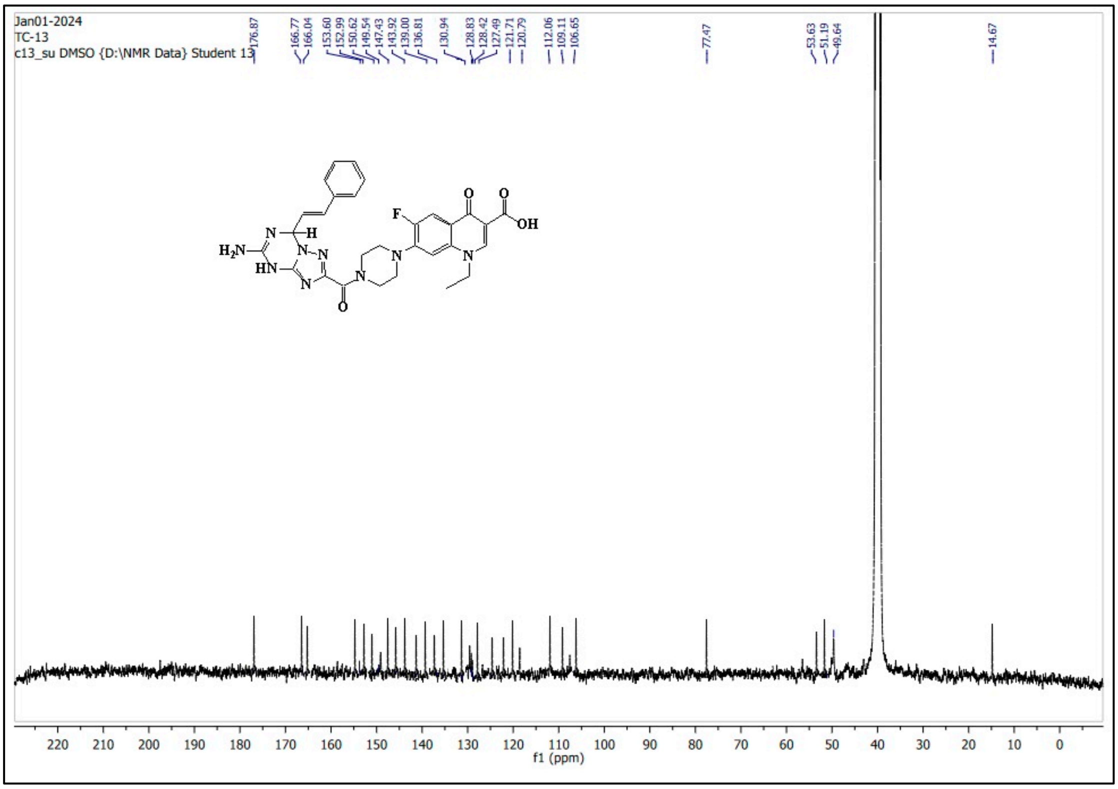

Figure S29: ^{13} CNMR Spectrum of compound **12**

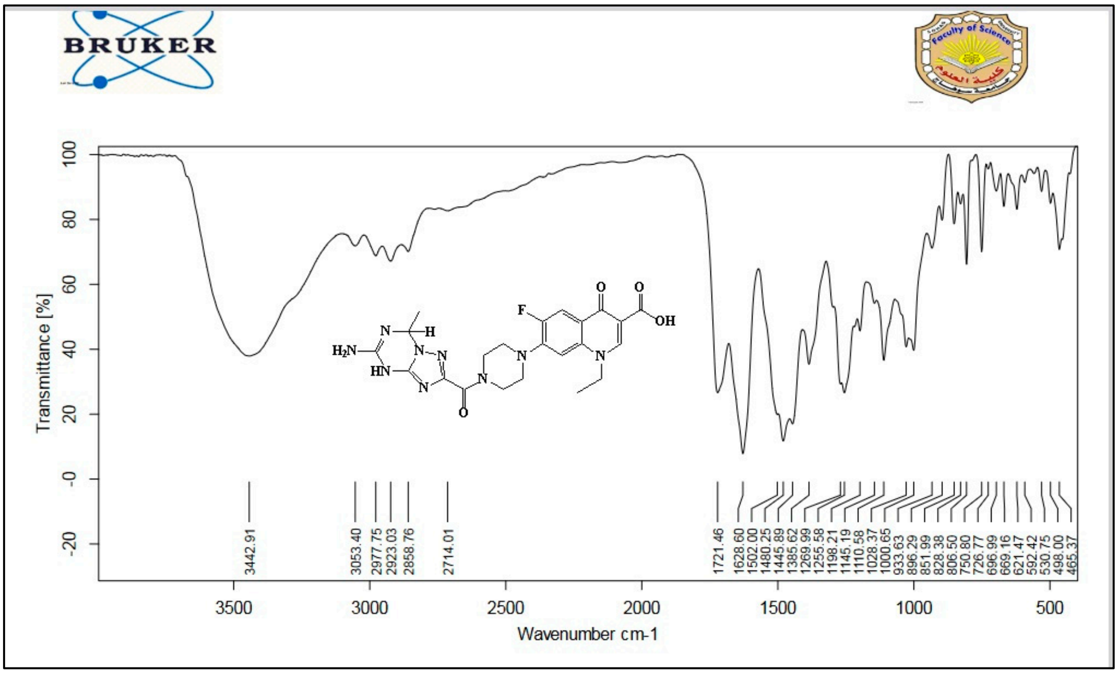

Figure S30: IR Spectrum of compound **13**

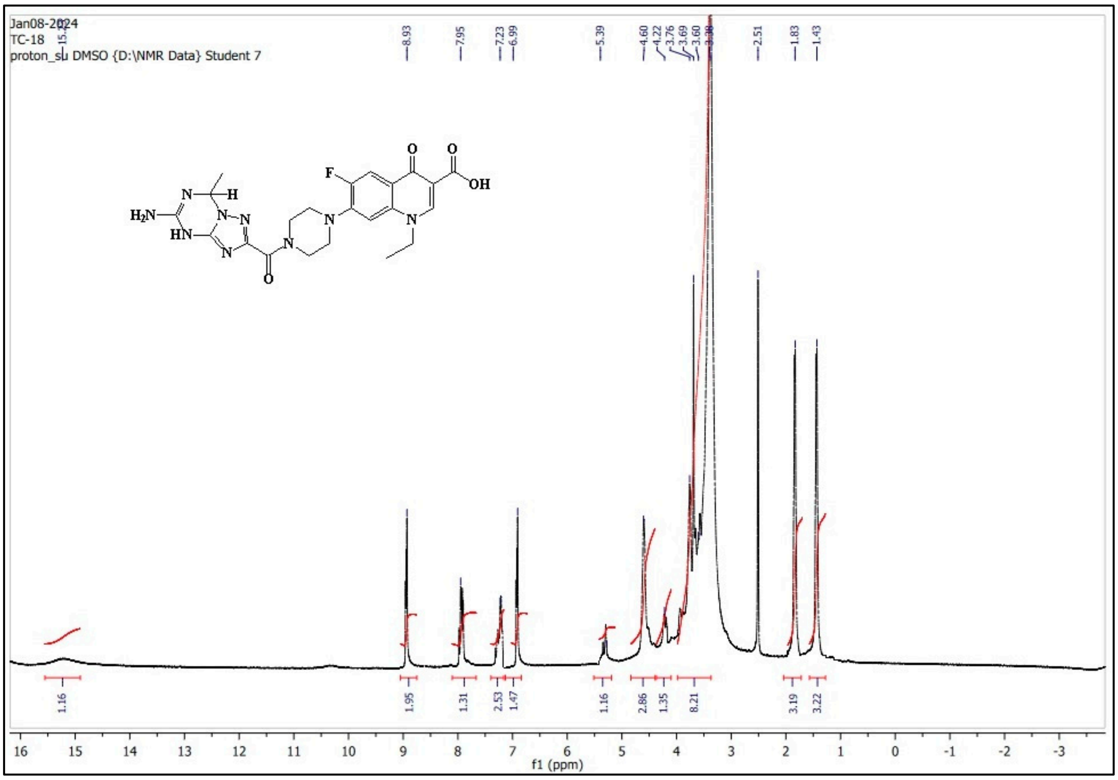

Figure S31: [1] HNMR Spectrum of compound **13**

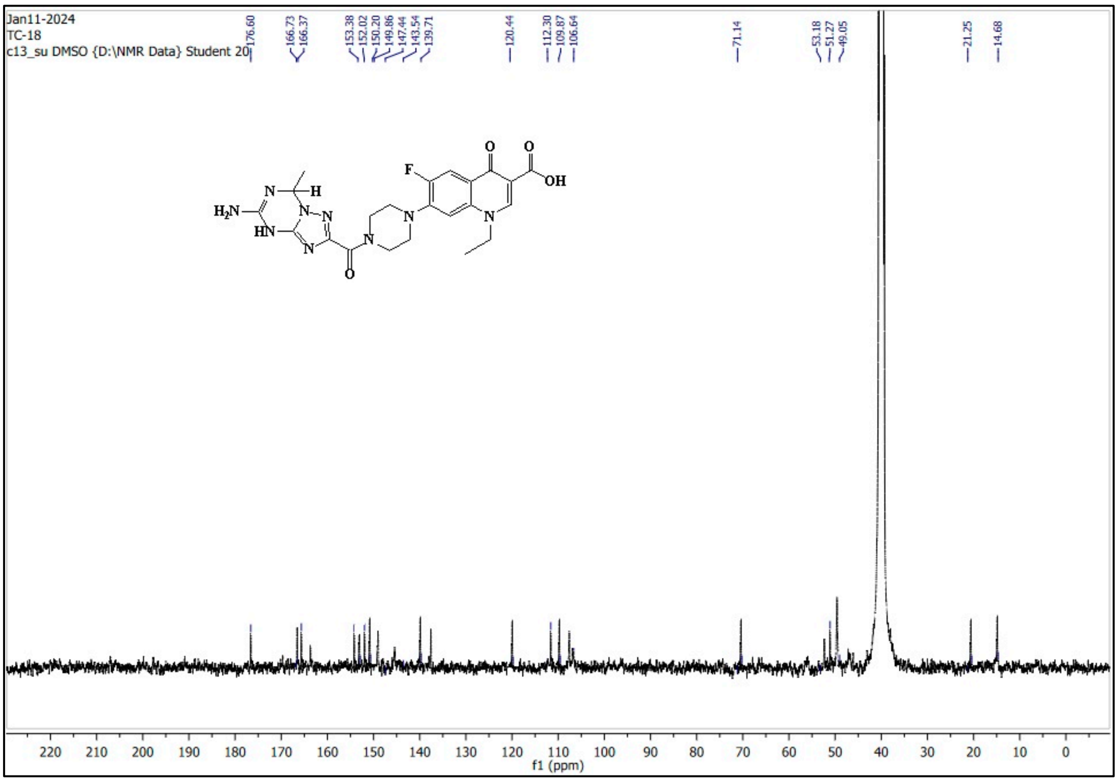

Figure SS32: [13] CNMR Spectrum of compound **13**

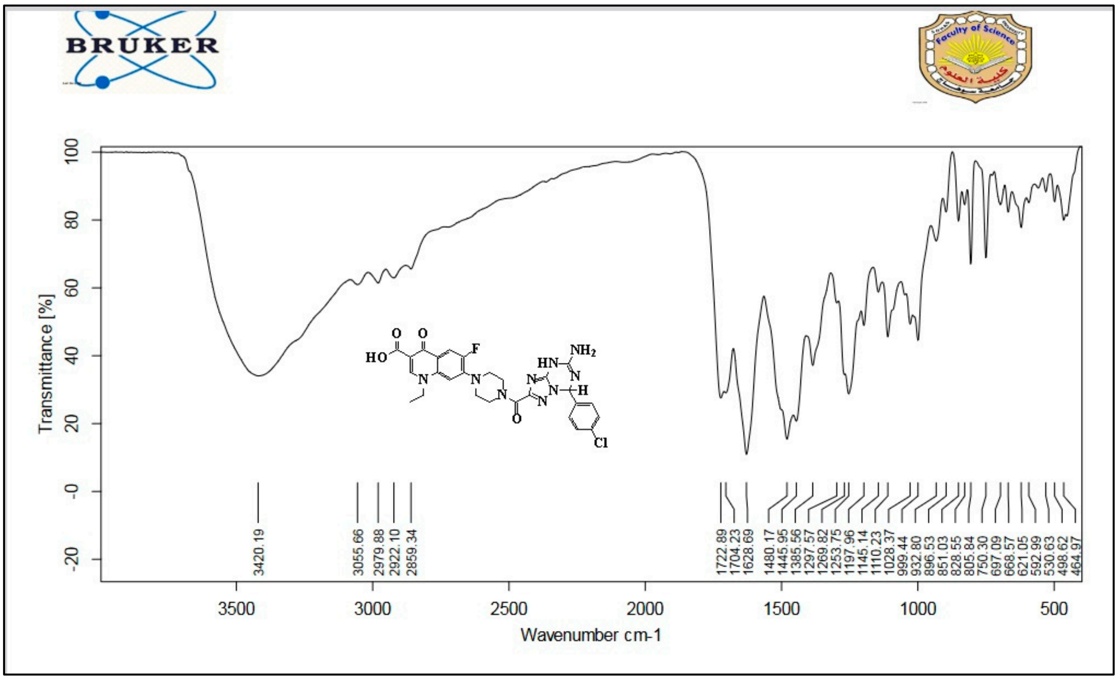

Figure S33: IR Spectrum of compound **14**

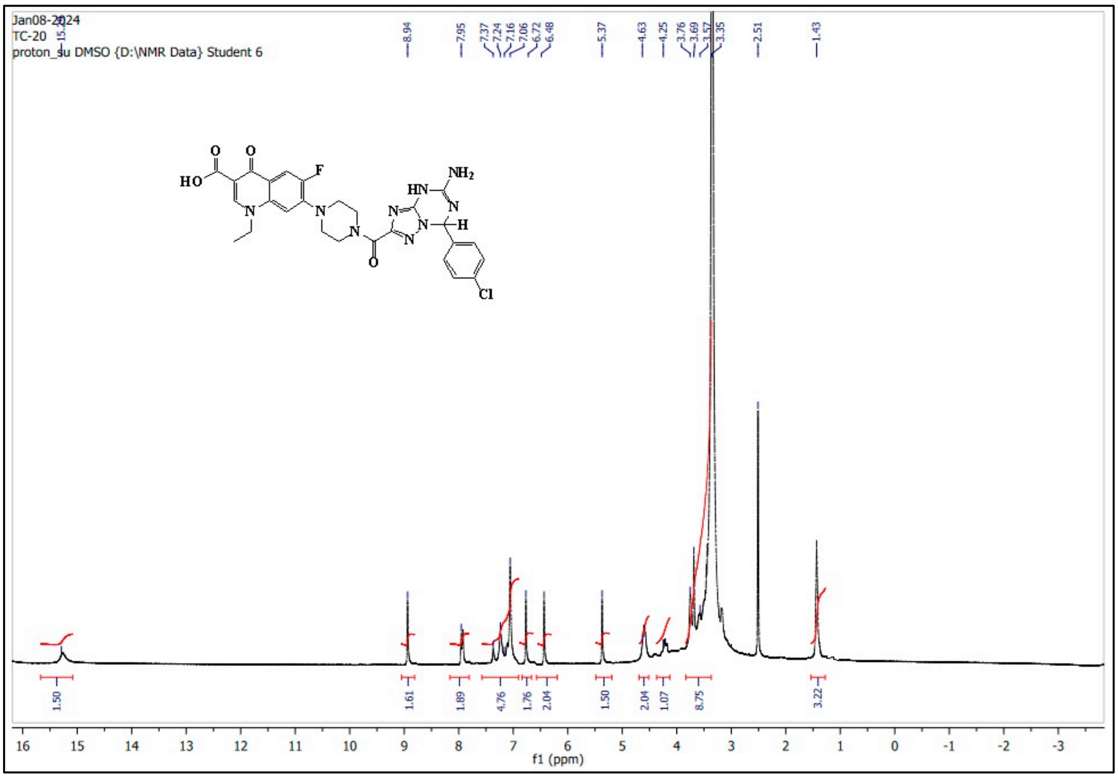

Figure S34: [1] HNMR Spectrum of compound **14**

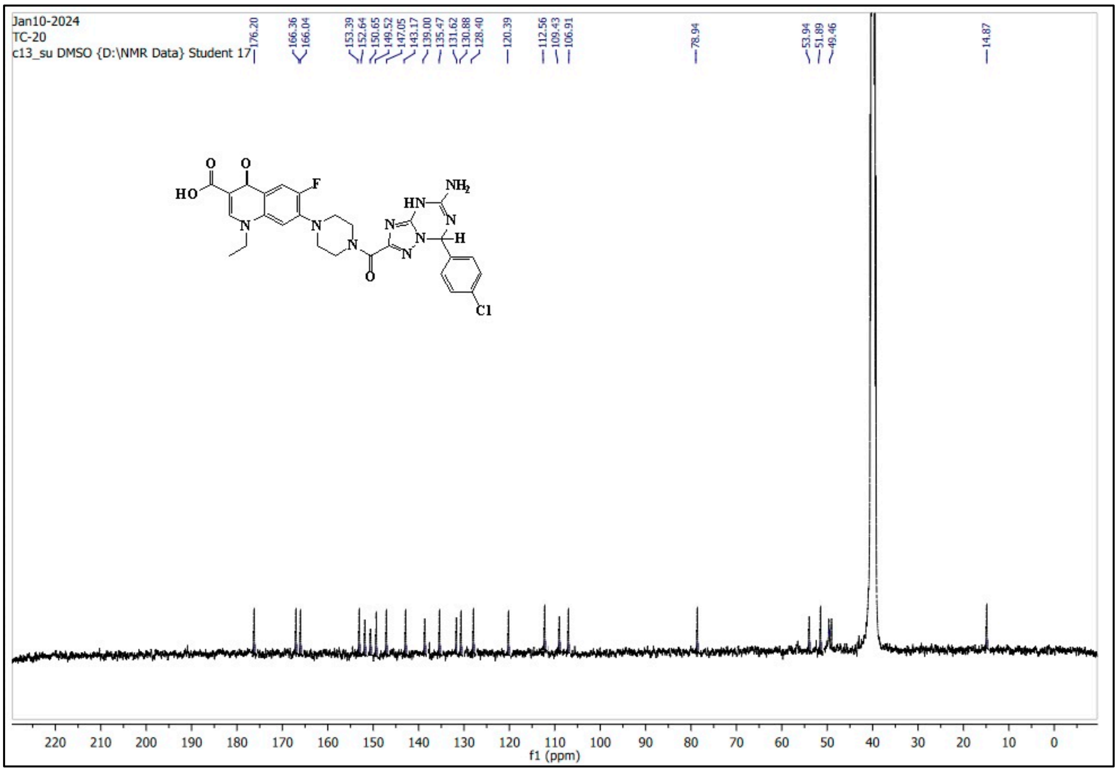

Figure SS35: [13] CNMR Spectrum of compound **14**

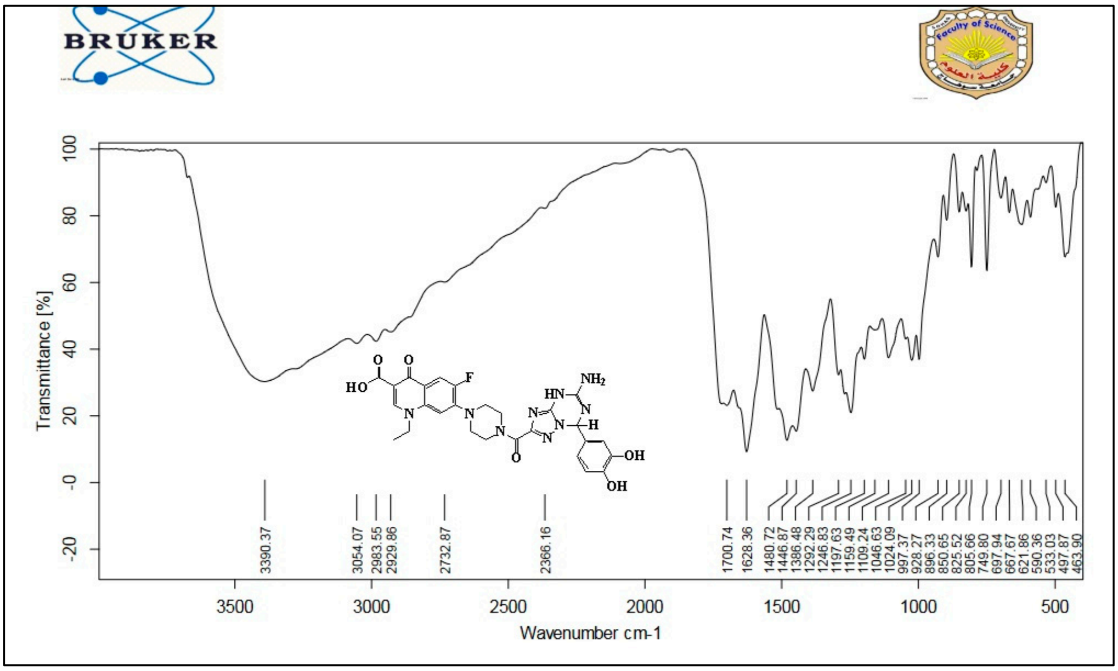

Figure S36: IR Spectrum of compound **15**

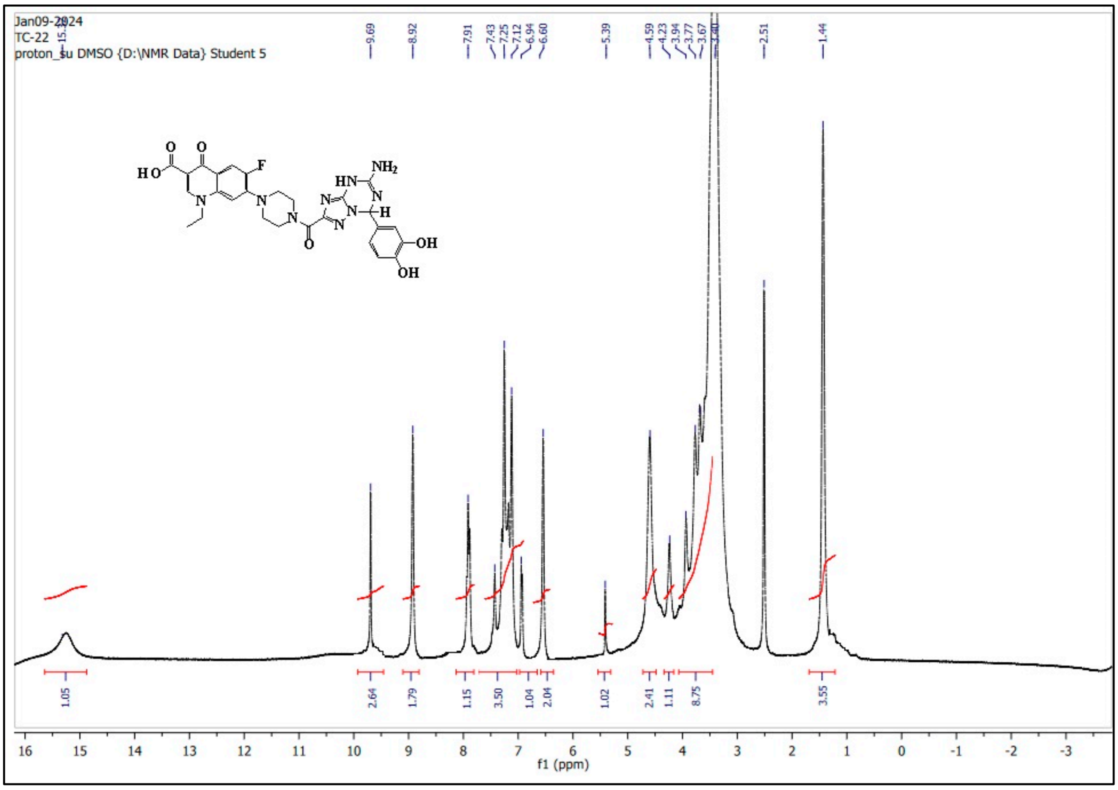

Figure S37: [1] HNMR Spectrum of compound **15**

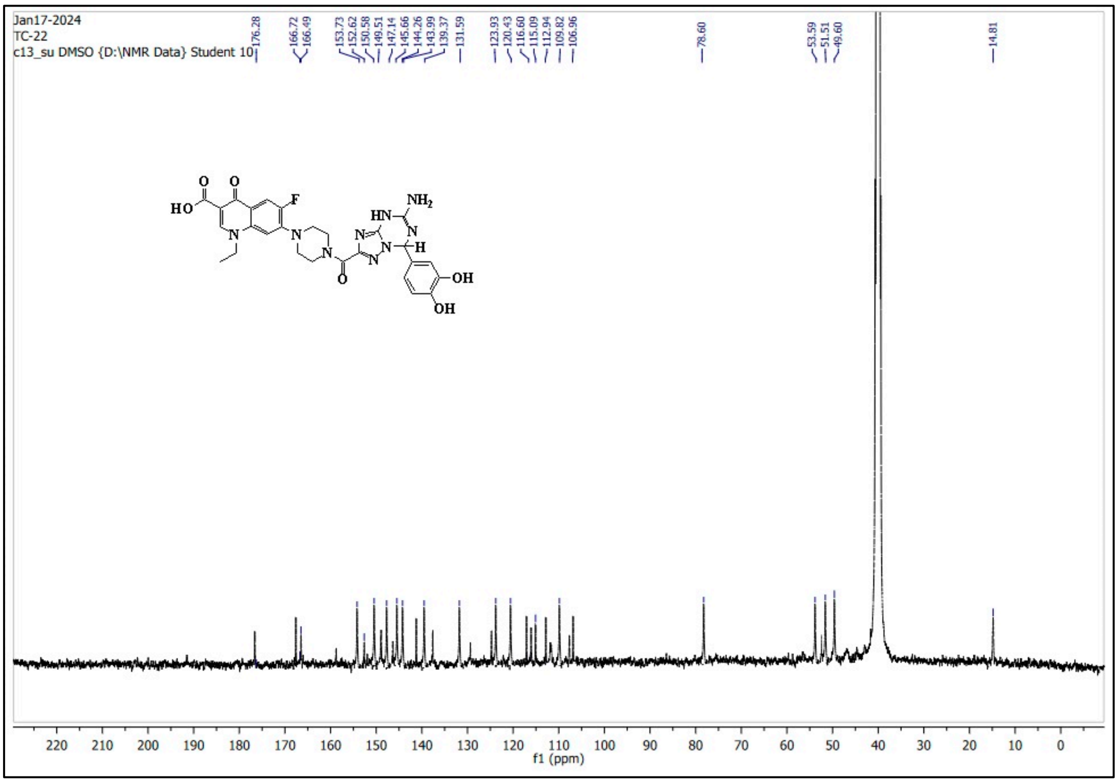

Figure S38: [13] CNMR Spectrum of compound **15**

Biology

Cytotoxicity results against normal skin cell BJ (detailed results)

researcher	assay	Date	cells
Dr.Hossam Aziz	MTT	31-Aug	BJ

	Blank	CC	Sample No. 15/BJ					Sample No. 14/BJ				
	1	2	3	4	5	6	7	8	9	10	11	12
A	B	C	100uM	25uM	6.3uM	1.6uM	0.4uM	100uM	25uM	6.3uM	1.6uM	0.4uM
B	B	C	100uM	25uM	6.3uM	1.6uM	0.4uM	100uM	25uM	6.3uM	1.6uM	0.4uM
C	B	C	100uM	25uM	6.3uM	1.6uM	0.4uM	100uM	25uM	6.3uM	1.6uM	0.4uM

ROBONIK P2000 Eia reader

Wave length: 570 nm

Reference: 630 nm

	1	2	3	4	5	6	7	8	9	10	11	12
A	0.001	0.477	0.209	0.257	0.326	0.387	0.439	0.187	0.246	0.295	0.364	0.432
B	0.001	0.435	0.221	0.262	0.339	0.364	0.455	0.193	0.257	0.327	0.372	0.443
C	0.001	0.459	0.197	0.271	0.322	0.377	0.441	0.202	0.262	0.316	0.355	0.452
mean	0.0004	0.457	0.209	0.263	0.329	0.376	0.445	0.194	0.255	0.3127	0.3637	0.4423
%		`	45.733	57.62	71.991	82.276	97.374	42.451	55.8	68.417	79.577	96.791

15/BJ

log conc.	% viability
2	45.733
1.3979	57.622
0.7959	71.991
0.1931	82.276
-0.4089	97.374

IC50=

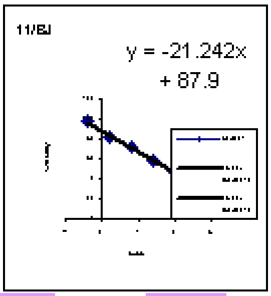

14/BJ

log conc.	% viability
2	42.45
1.3979	55.8
0.7959	68.42
0.1931	79.58
-0.409	96.79

IC50=

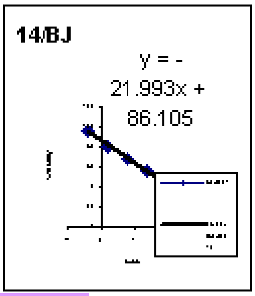

	Blank	CC	Sample No.			CPT/BJ		Sample No.		Dox/Caco2		
	**1**	**2**	**3**	**4**	**5**	**6**	**7**	**8**	**9**	**10**	**11**	**12**
A	B	C	100uM	25uM	6.3uM	1.6uM	0.4uM					
B	B	C	100uM	25uM	6.3uM	1.6uM	0.4uM					
C	B	C	100uM	25uM	6.3uM	1.6uM	0.4uM					

ROBONIK P2000 Eia reader

Wave length: 570 nm

Reference: 630 nm

	**1**	**2**	**3**	**4**	**5**	**6**	**7**	**8**	**9**	**10**	**11**	**12**
A	0.001	0.449	0.176	0.241	0.297	0.367	0.434					
B	0.001	0.483	0.181	0.237	0.312	0.358	0.449					
C	0.001	0.463	0.167	0.228	0.303	0.364	0.451					
mean	0.001	0.465	0.1747	0.235	0.304	0.363	0.4447	0	0	0	0	0
% viability			37.563	50.61	65.376	78.065	95.627	0	0	0	0	0

CPT/BJ

log conc.	% viability
2	37.563
1.3979	50.609
0.7959	65.376
0.1931	78.065
-0.4089	95.627

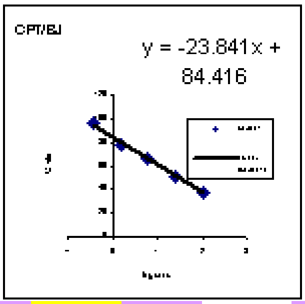

IC50=

log conc.	% viability
2	0
1.3979	0
0.7959	0
0.1931	0
-0.409	0

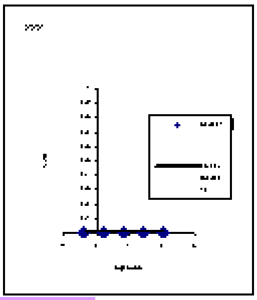

IC50=

Topoisomerase Assay (Detailed results)

TOPO I

code	IC50	conc	log	% inh
14		100	2	90
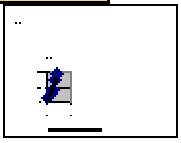		50	1.7	75
		25	1.4	43
		10	1	24
		1	0	13
EC				0
	[redacted]			

code	IC50	conc.uM	log conc	%inh
CPT		100	2	97
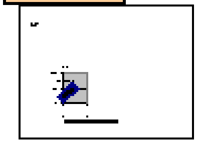		50	1.7	93
		25	1.4	78
		10	1	60
		1	0	42
EC				0
	[redacted]			

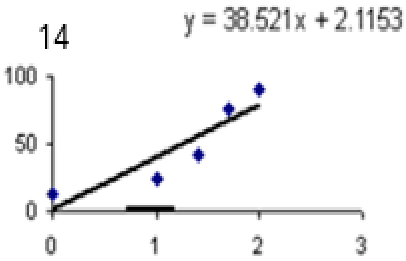

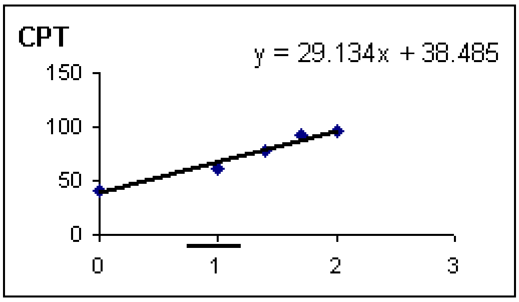

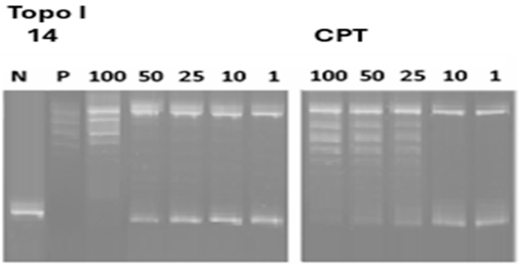

Topoisomerase II (detailed results)

TOPO II

code	IC50	conc	log	%inh
s14		100	2	88
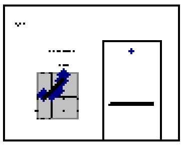		50	1.7	63
		10	1	21
		1	0	7.9
		0.1	-1	4.7
EC				0
	[redacted]			

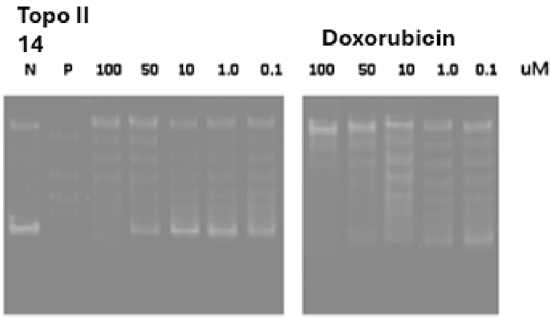

code	IC50	conc	log	%inh
Dox		100	2	92
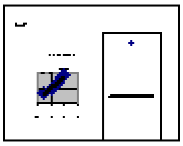		50	1.7	85
		10	1	65
		1	0	47
		0.1	-1	31
EC				0
	[redacted]			

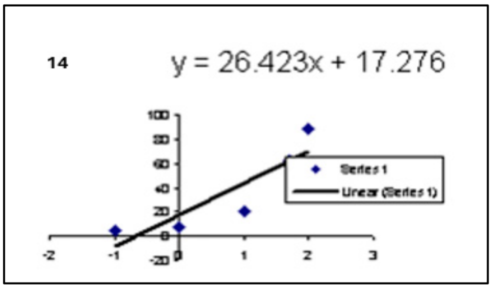

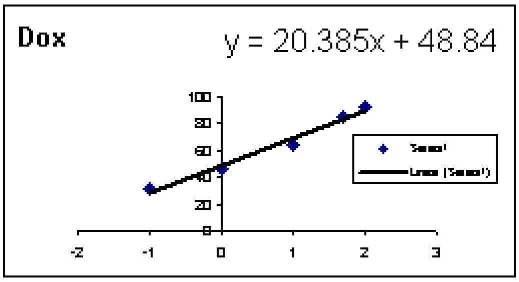

Evaluation of Topoisomerase I/II inhibition

The target compound **14** showed potent anticancer activity was selected for topoisomerases inhibition assay in comparison to untreated positive controls using the Elisa kit of human DNA topoisomerase following the described protocols. Compounds 14 was evaluated for topoisomerase Iα and topoisomerase IIβ inhibitory activity utilizing the human DNA topoisomerase Elisa kit.

Two folds of serial dilution was accomplished after standards, and the tested compound were dissolved in sample diluent. Horseradish peroxidase (HRP-avidin) and biotin-conjugated antibody were diluted 10 times each. Each well received 100 µL of each concentration of the standard or test chemicals, which were then added and incubated at 37 °C for 60 min. After each well's liquid had been carefully removed, 100 µL of a solution of biotin-conjugated antibody had been added, and each well had been incubated for 1 h at 37 °C. The microtiter plate was rinsed three times and given room to aspirate. After that, the plate was incubated at 37 °C for 60 min with 100 µL of HRP-avidin solution added to each well. After that, the plate was aspirated and cleaned 5 times. Each well received 90 µL of TMB substrate, and the plate was incubated for 30 min. at 37 °C in a light-protected environment. Finally, stop solution (50 µL) was loaded, and within 5 minutes, the absorbance at 450 nm was measured spectrophotometrically.

Cycle analysis and measurement of apoptotic potential

The effect of compound **14** on cell cycle progression of Skin A431 cell line was evaluated using Propidium Iodide Flow Cytometry Kit to measure the DNA content according to the reported protocols.

A431 cells used in this work were obtained from the American Type Culture Collection. Cells were cultured in DMEM (Invitrogen/Life Technologies) supplemented with 10% FBS (Hyclone), 10 µg/ml insulin (Sigma), and 1% penicillin-streptomycin. The remaining chemicals and reagents were all from Sigma or Invitrogen. Growth media was removed, cells washed with warm PBS and aspirated. Adhered cells were detached from media by addition of 5 ml 10% trypsin/EDTA and incubated 2 minutes in the incubator. The suspended cells harvested, aspirated to a falcon tube, centrifuged at 2000 rpm for 5 minutes. Cell count performed after suspending the cells into a fresh media and stained with trypan blue, counted and used for preparation of the appropriate number of cells.

A431 cells were treated by the IC_{50} concentration of compound **14** (76.7 µM. The influence -of compound **14** to induce apoptosis in A431 cell line was estimated in comparison to the untreated cells as negative control. Following the manufacturer's instructions, the Annexin V-FITC Apoptosis Detection Kit (Bio Vision Research Products, USA) was used to analyze cell apoptosis. Briefly, 500 µL of 1X Binding

buffer was used to resuspend 1-5x10^5 cells that had been harvested by centrifugation. Propidium iodide (PI, 50 mg/ml) and Annexin V-FITC, each administered in 5 μL, were also added. The cells were first incubated for 5 minutes at room temperature in the dark before being analyzed using the Annexin V-FITC binding flow cytometric technique (Ex=488 nm; Em =530 nm) with a FITC signal detector (typically FL1) and PI staining with a phycoerythrin emission signal detector (typically FL2). Before exposing adherent cells to Annexin V-FITC, we gently trypsinized and gave them a single wash in serum-containing medium (A.3-5). Cell synchronisation is done using Double-thymidine synchronization method and the used Media is double modified eagle media (DMEM).
